# Laterally Oriented Dendritic Passivation via In Situ Zn Reconstruction for Stabilizing NiMo Catalysts under Dynamic Electrolysis

**DOI:** 10.1002/advs.202520103

**Published:** 2026-01-12

**Authors:** Taeyoung Jeong, Hyun‐Jae Park, Myeongjin Kim

**Affiliations:** ^1^ School of Energy Engineering Kyungpook National University Daegu Republic of Korea

**Keywords:** dendrite, hydrogen evolution reaction, load fluctuation, NiMo, sacrificial species

## Abstract

Integrating water electrolyzers with intermittent renewable energy poses critical durability challenges from dynamic load fluctuations inducing catalyst degradation. We report a zinc‐mediated sacrificial protection strategy enhancing NiMo catalyst stability through in situ dendritic passivation. Zinc‐decorated NiMo on nickel felt (Zn‐NiMo/NF) exhibits considerable hydrogen evolution activity (94.6 mV overpotential at 50 mA cm^−2^) comparable to Pt/C. Under stringent load fluctuation cycling protocols (−500/50 mA cm^−2^), the zinc overlayer spontaneously reconstructs into laterally oriented, NiMo‐enriched dendrites providing dual protection: physical barriers suppressing dissolution (order‐of‐magnitude reductions in metal leaching) and sacrificial buffering wherein zinc preferentially oxidizes to zincate, shielding nickel from irreversible hydroxide formation. Zn‐NiMo/NF maintains stable performance while pristine NiMo/NF degrades substantially. Anion exchange membrane electrolyzer validation confirms minimal voltage escalation over 100 h cycling (1.645– 1.667 V), outperforming Pt/C (1.7028–1.857 V). This establishes sacrificial interface engineering as an effective paradigm for robust earth‐abundant electrocatalysts in renewable energy‐integrated hydrogen production.

## Graphical abstract

This study highlights the effectiveness of Zn‐induced dendrite layers in enhancing the durability of NiMo HER catalysts under dynamic electrochemical conditions. Through in situ dendritic passivation, the Zn‐NiMo catalyst preserves catalytic active sites and mitigates irreversible Ni oxidation/hydroxylation during repeated load fluctuation.

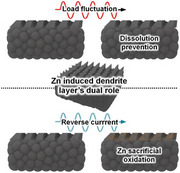

## 1 Introduction

Anion exchange membrane water electrolyzers (AEMWEs) have emerged as a compelling platform for sustainable hydrogen production, operating in alkaline environments with earth‐abundant catalysts and thereby circumventing reliance on precious metals [1, 2]. Within AEMWE architectures, catalyst‐coated substrates (CCSs) have garnered attention for practical implementation owing to their superior mechanical robustness, straightforward fabrication, and enhanced mass transport characteristics compared to catalyst‐coated membranes (CCMs) [3, 4]. CCS electrodes are particularly amenable to electrodeposition‐based synthesis, enabling uniform coverage, conformal coating on 3D porous substrates, and scalability to industrially relevant dimensions [5, 6]. Nickel‐based porous architectures, including nickel foam and nickel felt, represent the predominant substrate materials due to their exceptional electrical conductivity and corrosion resistance in alkaline media [7, 8]. However, pristine nickel exhibits suboptimal hydrogen evolution reaction (HER) activity arising from non‐ideal hydrogen binding energetics [9]. Alloying nickel with molybdenum has been extensively pursued to address this limitation, as molybdenum incorporation modulates the electronic structure of nickel through ligand effects and electronic coupling that shift the d‐band center closer to the Fermi level, thereby optimizing hydrogen adsorption free energy while introducing synergistic active sites at Ni─Mo heterointerfaces [10, 11, 12, 13]. Consequently, NiMo‐based alloys have established themselves as among the most promising non‐noble HER electrocatalysts, consistently delivering high catalytic activity while maintaining compatibility with scalable electrodeposition techniques [14].

Despite these favorable attributes, practical deployment of NiMo catalysts in renewable energy‐integrated AEMWEs confronts substantial challenges from inherently dynamic operational profiles imposed by intermittent power sources [15, 16]. Real‐world electrolyzers interfaced with solar or wind energy rarely operate under steady‐state conditions but instead experience severe load fluctuations driven by diurnal cycles, weather variability, and grid demand dynamics [17, 18]. Under such operation, catalysts encounter multifaceted electrochemical stresses far exceeding those in conventional constant‐current laboratory protocols, including abrupt current density transitions, transient zero‐current intervals, and potentially reverse‐current events during grid stabilization [19]. These dynamic conditions activate multiple degradation pathways: repeated polarity reversals induce structural reorganization and mechanical stress at the catalyst–substrate interface; nickel sites undergo progressive oxidation to catalytically inactive β‐Ni(OH)_2_ or γ‐NiOOH phases during open‐circuit intervals or anodic excursions; molybdenum species experience partial dissolution under oxidative conditions [20, 21, 22]. Collectively, these mechanisms erode active sites, introduce charge‐transfer impedances through insulating oxide formation, and compromise long‐term durability. Furthermore, electrodeposited catalyst layers lacking polymeric binders exhibit heightened susceptibility to delamination under repeated mechanical stress from dynamic cycling and gas bubble evolution [23, 24, 25].

To mitigate degradation under dynamic stresses, sacrificial protection strategies inspired by corrosion science offer an elegant solution wherein a component with more negative standard electrode potential undergoes preferential oxidation, electrochemically shielding the active framework [26, 27]. Zinc presents compelling advantages with its substantially negative potential (*E*° = −0.76 V vs SHE) relative to nickel (*E*° = −0.25 V vs SHE), protecting the underlying NiMo phase [28, 29]. However, zinc's propensity for dendritic growth during cycling—conventionally regarded as detrimental in battery systems—could instead be harnessed as a beneficial protective feature [30, 31, 32]. Herein, we report zinc‐decorated NiMo catalysts on nickel felt (Zn‐NiMo/NF) demonstrating exceptional stability under severe load fluctuation. Comprehensive characterization reveals the initially conformal zinc overlayer undergoes spontaneous in situ reconstruction into laterally oriented, NiMo‐enriched dendritic architectures during dynamic cycling, forming a protective layer that suppresses physical dissolution while mitigating irreversible oxidation through sacrificial zinc oxidation to zincate species. The Zn‐NiMo/NF catalyst maintains stable performance under stringent protocols alternating between −500 and +50 mA cm^−2^ while pristine NiMo/NF exhibits pronounced degradation. Single‐cell AEMWE validation confirms practical viability with minimal voltage escalation (1.645 to 1.667 V at 1.0 A cm^−2^ over 100 h) compared to severe degradation in Pt/C systems. This work establishes sacrificial interface engineering with in situ dendritic reconstruction as an effective strategy for stabilizing earth‐abundant HER catalysts in dynamic renewable energy‐integrated electrolysis environments.

## 2 Results and Discussion

**FIGURE 1:**
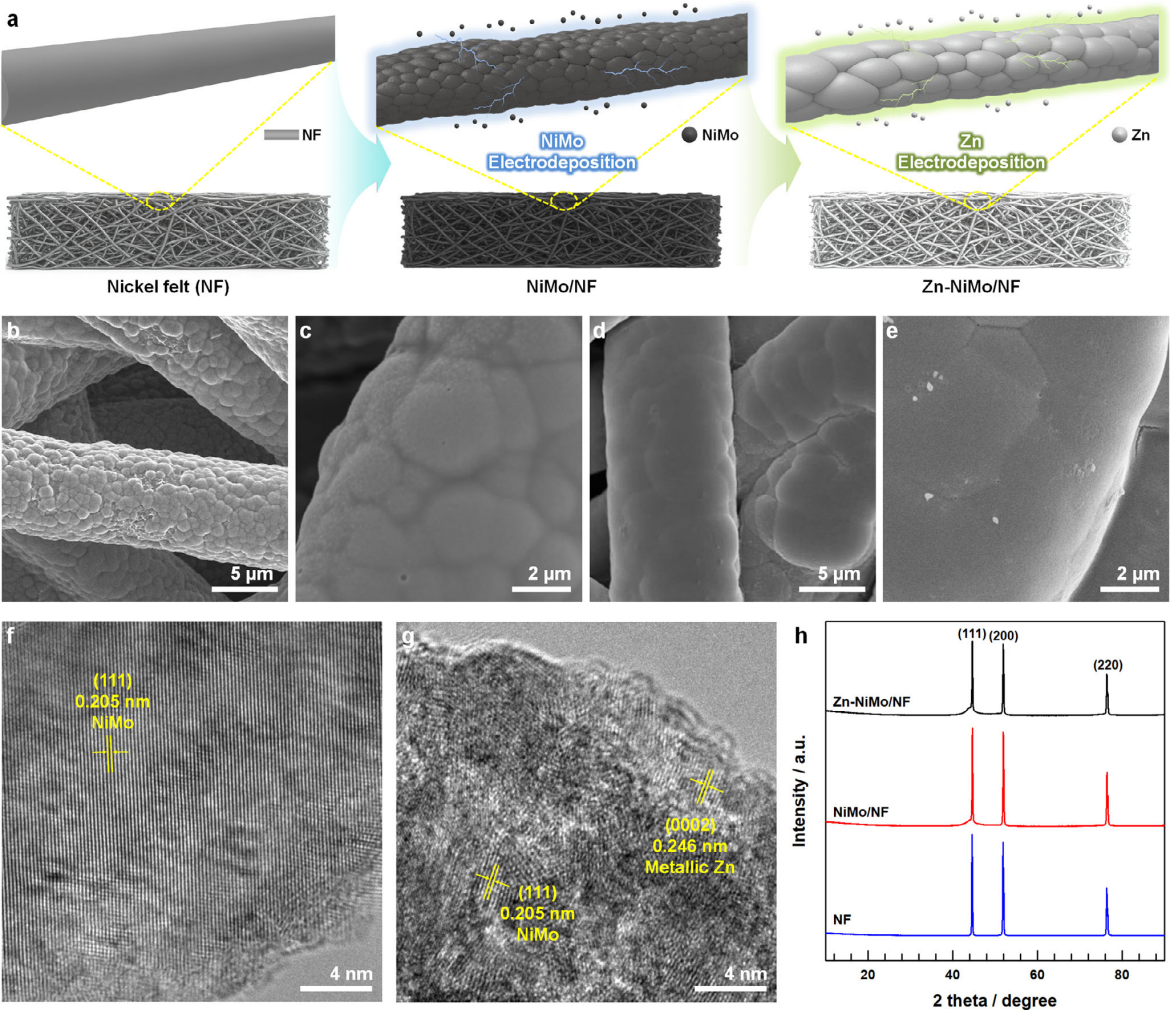
a) Schematic scheme of fabricating NiMo alloy and Zn‐NiMo alloy on nickel felt (NF) using electrodeposition method. Field emission scanning electron microscopy (FE‐SEM) images of b‐c) NiMo/NF and d‐e) Zn‐NiMo/NF at different magnifications. High‐resolution transmission electron microscopy (HR‐TEM) images of f) NiMo/NF and g) Zn‐NiMo/NF. h) X‐ray diffraction (XRD) patterns of Zn‐NiMo/NF, NiMo/NF, and NF.

The preparation of NiMo alloys and zinc‐modified NiMo alloys (Figure 1a) was conducted via electrodeposition techniques employing nickel felt as the conductive substrate (NF). The fabrication protocol commenced with the electrochemical deposition of a NiMo alloy layer onto the nickel felt substrate (NiMo/NF) through controlled immersion in an aqueous bath containing appropriate nickel and molybdenum precursor compounds. Following this initial deposition, zinc incorporation was achieved by subjecting the NiMo/NF electrode to a secondary electrodeposition process in a zinc‐containing electrolyte, ultimately producing the zinc‐decorated NiMo catalyst (Zn‐NiMo/NF). The morphological characteristics of NiMo/NF and Zn‐NiMo/NF catalysts were systematically examined using field‐emission scanning electron microscopy (FE‐SEM). FE‐SEM micrographs of the pristine NiMo/NF catalyst (Figure 1b,c) reveal a uniformly smooth and continuous morphology featuring a conformal alloy layer that intimately coats the nickel felt substrate (Figure S1). The surface topology exhibits a characteristic nodular architecture comprising interconnected nanoscale grains, consistent with a nucleation‐dominated growth mechanism. Conversely, the Zn‐NiMo/NF catalyst (Figure 1d,e) demonstrates a comparatively smoother surface texture accompanied by enlarged particle dimensions and more pronounced grain boundaries. This morphological evolution reflects a densification and grain coarsening mechanism intrinsically linked to voltage‐induced nucleation phenomena [33, 34]. During Zn electrodeposition, the applied electrochemical potential effectively diminishes the nucleation energy barrier, thereby promoting instantaneous nucleation that facilitates lateral grain impingement and coalescence of the underlying NiMo nanocrystallites, ultimately yielding a densified, continuous NiMo framework [35, 36].

High‐resolution transmission electron microscopy (HR‐TEM) analysis (Figure 1f,g) corroborates these observations, revealing that both NiMo/NF and Zn‐NiMo/NF exhibit identical d‐spacing values of 0.205 nm, which corresponds to the (111) crystallographic plane of the face‐centered cubic (FCC) NiMo phase [37]. This lattice parameter demonstrates excellent concordance with the (111) spacing of metallic nickel, confirming that the NiMo alloy retains the characteristic FCC crystal structure inherent to metallic Ni [38]. Additionally, the Zn‐NiMo/NF catalyst displays a distinct d‐spacing of 0.246 nm, attributable to the (0002) plane of hexagonal close‐packed metallic zinc [39, 40]. These structural findings are further substantiated by X‐ray diffraction (XRD) analysis (Figure 1h), which reveals characteristic Bragg reflections corresponding to the (111), (200), and (220) planes of FCC‐Ni consistently across the pristine nickel felt, NiMo/NF, and Zn‐NiMo/NF [37, 41]. The subtle shoulder peak adjacent to the (111) reflection serves as a diagnostic signature of the NiMo alloy phase. Notably, the absence of distinct zinc diffraction peaks can be attributed to the surface‐confined and ultrathin nature of the zinc overlayer, as XRD predominantly interrogates bulk crystalline phases rather than surface‐localized species. To definitively establish the metallic nature of both NiMo and Zn phases, complementary Raman spectroscopy was employed (Figure S2). The absence of discernible Raman signatures in both NiMo/NF and Zn‐NiMo/NF samples is consistent with the expected behavior of metallic phases, wherein delocalized conduction electrons and the paucity of polarizable vibrational modes preclude the observation of characteristic Raman bands, thereby confirming the predominantly metallic character of the constituent phases [42, 43].

**FIGURE 2:**
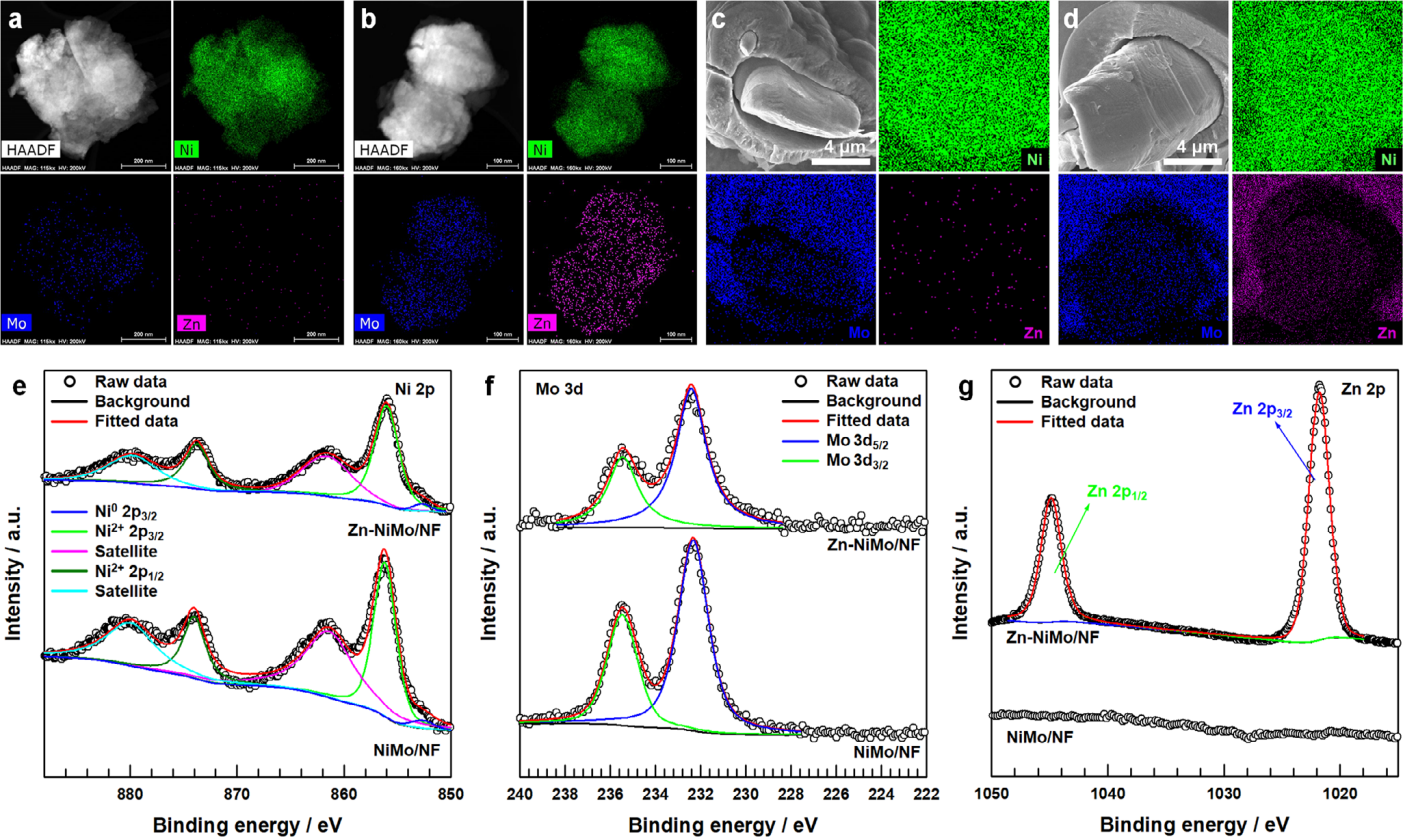
High angle annular dark‐field scanning TEM (HAADF‐STEM) images with corresponding energy‐dispersive X‐ray spectroscopy (EDS) elemental mapping images of Ni, Mo, Zn elements of a) NiMo/NF and b) Zn‐NiMo/NF. SEM image at edge view with corresponding EDS elemental mapping images of Ni, Mo and Zn elements of c) NiMo/NF and d) Zn‐NiMo/NF electrode. X‐ray photoelectron spectroscopy (XPS) e) Ni 2p spectra, f) Mo 3d, and g) Zn 2p spectra of Zn‐NiMo/NF and NiMo/NF.

To validate the uniform deposition of the NiMo layer and confirm successful zinc incorporation, comprehensive elemental distribution analysis was conducted using energy‐dispersive X‐ray spectroscopy (EDS) coupled with scanning transmission electron microscopy (STEM). The resulting EDS elemental maps demonstrate homogeneous spatial distribution of nickel and molybdenum throughout the NiMo alloy matrix (Figure 2a and Figure S3a,), while simultaneously confirming the uniform dispersion of zinc, nickel, and molybdenum across the Zn‐NiMo/NF composite (Figure 2b and Figure S3b). Quantitative compositional analysis via the inductively coupled plasma optical emission spectrometry (ICP‐OES) revealed that the NiMo catalyst comprised 86.6 wt% Ni and 13.4 wt% Mo, whereas the Zn‐modified catalyst exhibited a composition of 82.6 wt% Ni, 12.8 wt% Mo, and 4.6 wt% Zn (Table S1). Cross‐sectional SEM micrographs of both NiMo/NF and Zn‐NiMo/NF specimens (Figure 2c,d) provide corroborative evidence for the formation of a well‐defined NiMo alloy overlayer on the nickel felt substrate, followed by subsequent zinc deposition atop the preformed NiMo matrix. The elemental mapping data reveal a stratified architectural motif wherein nickel exhibits homogeneous distribution throughout the substrate, molybdenum demonstrates preferential enrichment within the intermediate coating layer, and zinc shows pronounced accumulation at the outermost surface region. This compositional gradient unambiguously confirms the successful conformal deposition of zinc onto the underlying NiMo surface, establishing a hierarchical multilayer architecture that preserves the structural integrity of each constituent phase while enabling synergistic interactions at the heterointerfaces.

X‐ray photoelectron spectroscopy (XPS) was employed to elucidate the electronic structure and oxidation states of nickel and molybdenum within both NiMo/NF and Zn‐NiMo/NF catalysts. The high‐resolution Ni 2p XPS spectra (Figure 2e) reveal two predominant photoelectron peaks centered at 856.0 and 873.7 eV, assignable to the Ni 2p_3/2_ and Ni 2p_1/2_ core levels, respectively, accompanied by characteristic satellite features and a minor metallic nickel component at 852.2 eV. These primary photoelectron signatures are diagnostic of divalent nickel (Ni^2+^) species, substantiating the presence of surface‐oxidized nickel phases within the NiMo alloy matrix [44]. Concurrently, the Mo 3d core‐level spectra (Figure 2f) exhibit two distinct photoelectron peaks positioned at 232.3 and 235.4 eV, corresponding to the Mo 3d_5/2_ and Mo 3d_3/2_ spin–orbit components, respectively. These binding energies are characteristic of hexavalent molybdenum (Mo^6+^), which represents the thermodynamically favored oxidation state under ambient atmospheric conditions and is routinely encountered in naturally occurring surface oxide phases [45]. Significantly, both NiMo/NF and Zn‐NiMo/NF demonstrate virtually identical peak positions and spectral profiles, conclusively establishing that zinc incorporation proceeds without perturbing the intrinsic oxidation states of the nickel and molybdenum constituents within the alloy framework. The Zn 2p core‐level region (Figure 2g) displays two well‐resolved photoelectron peaks at binding energies of 1021.8 and 1044.8 eV, attributed to the Zn 2p_3/2_ and Zn 2p_1/2_ transitions, respectively [46]. These spectroscopic signatures are consistent with divalent zinc (Zn^2+^) species, corroborating previous investigations of electrodeposited zinc systems and indicating the formation of a native zinc oxide overlayer through partial surface oxidation under ambient atmospheric exposure [47]. This observation confirms the expected chemical behavior wherein the outermost zinc layer undergoes spontaneous oxidation while preserving the underlying metallic character of the core structure.

**FIGURE 3:**
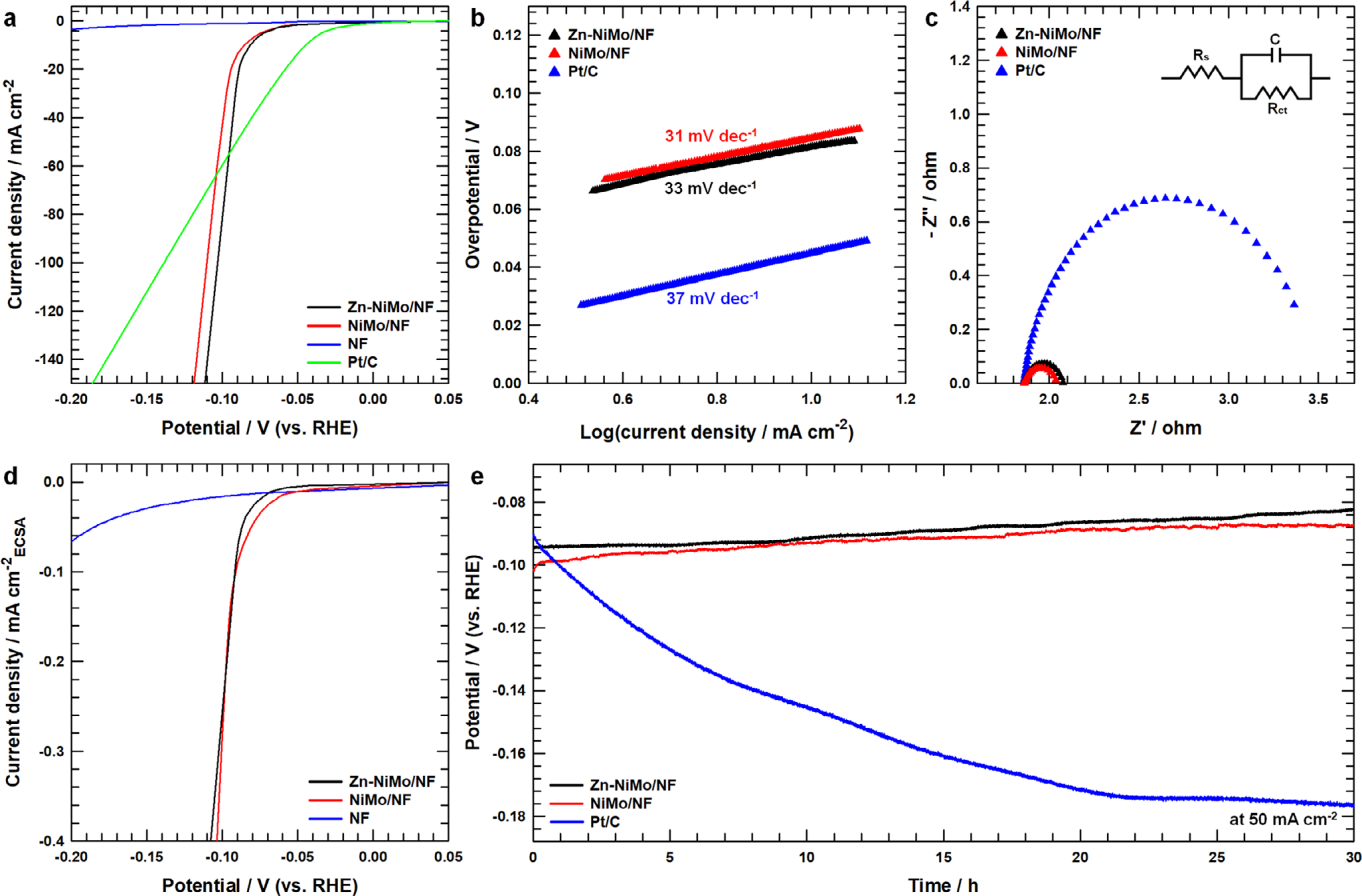
HER activity trend and long‐term stability test of catalysts under HER conditions. a) iR‐corrected Linear sweep voltammetry (LSV) curves of Zn‐NiMo/NF, NiMo/NF, NF, and Pt/C. b) Tafel plots for Zn‐NiMo/NF, NiMo/NF, and Pt/C. c) Nyquist plots for Zn‐NiMo/NF, NiMo/NF, and Pt/C. d) LSV curves of Zn‐NiMo/NF, NiMo/NF, and NF. Currents were normalized to ECSA after iR‐correction. e) Chronopotentiometric (CP) profile of Zn‐NiMo/NF, NiMo/NF, and Pt/C at a constant current density of 50 mA cm^−2^ for 30 h.

The HER catalytic performance of NiMo/NF and Zn‐NiMo/NF was systematically assessed under alkaline conditions using H_2_‐saturated 1 m KOH electrolyte. As illustrated in Figure 3a, linear sweep voltammetry (LSV) analysis reveals that both NiMo/NF and Zn‐NiMo/NF exhibit higher overpotentials relative to the commercial Pt/C benchmark within the low current density regime. However, their catalytic performance converges with and ultimately surpasses that of Pt/C at higher current densities, demonstrating considerable activity under industrially relevant operating conditions. Notably, the Zn‐NiMo/NF catalyst displays comparable HER activity, requiring an overpotential of 94.6 mV to achieve a current density of 50 mA cm^−2^, which represents a substantial improvement over bare nickel felt (307.7 mV at 50 mA cm^−2^) and exhibits performance metrics comparable to both Pt/C and NiMo/NF (90.6 mV and 101.5 mV at 50 mA cm^−2^, respectively). Comparative analysis (Table S2) confirms that the catalytic activity of Zn‐NiMo/NF is competitive with or exceeds that of state‐of‐the‐art HER electrocatalysts reported in the literature. Furthermore, Zn‐NiMo/NF demonstrates favorable reaction kinetics, as evidenced by a low Tafel slope of 33 mV dec^−1^ (Figure 3b) and minimal charge‐transfer resistance of 0.21 Ω (Figure 3c). These exceptionally low charge‐transfer resistance values observed for both NiMo/NF (0.19 Ω) and Zn‐NiMo/NF stem from the intrinsic metallic conductivity of the NiMo alloy framework, coupled with the advantageous binder‐free electrode architecture achieved through direct electrochemical growth on the conductive nickel substrate [48]. This integrated design effectively minimizes interfacial resistance and promotes facile electron transport kinetics. By contrast, conventional Pt/C catalysts inevitably introduce multiple interfacial junctions (Pt‐carbon‐binder‐substrate), each contributing additional contact and ionic resistances that impede overall electron transfer [49].

To rigorously evaluate the intrinsic catalytic activity independent of surface area effects, the electrochemical active surface area (ECSA) was determined through double‐layer capacitance (*C*_dl_) measurements derived from cyclic voltammetry (CV) experiments (Figure S4). The *C*_dl_ values were quantified as 6.094 mF cm^−2^ and 12.88 mF cm^−2^ for NiMo/NF and Zn‐NiMo/NF, respectively (Figure S5). The enhanced *C*_dl_ and correspondingly larger ECSA (Figure S6) exhibited by Zn‐NiMo/NF can be attributed to voltage‐driven nucleation phenomena and subsequent grain growth during zinc electrodeposition, which effectively increases the density of accessible catalytic sites. Importantly, following ECSA normalization (Figure 3d), the intrinsic catalytic performance of NiMo/NF becomes virtually indistinguishable from that of Zn‐NiMo/NF, indicating that the apparent activity enhancement observed for the zinc‐modified catalyst originates predominantly from the increased electrochemically accessible surface area introduced during zinc electrodeposition, rather than intrinsic activity improvement. In addition, the nearly identical Tafel slopes provide additional evidence that Zn incorporation does not alter the intrinsic HER kinetics. This convergence of intrinsic activity can be rationalized by considering the relatively low zinc content (4.6 wt%) and its highly uniform, conformal distribution across the NiMo surface. Such a thin and homogeneous zinc overlayer is unlikely to substantially occlude the underlying NiMo active sites or introduce significant kinetic barriers to charge transfer (Figure 3c), thereby preserving the inherent catalytic properties of the base NiMo alloy while simultaneously augmenting the total number of active sites through morphological modification. Long‐term electrochemical stability was rigorously evaluated through chronopotentiometry (Figure 3e) conducted at a constant cathodic current density of −50 mA cm^−2^ over a 30 h operational period. Both NiMo/NF and Zn‐NiMo/NF catalysts exhibited nearly equivalent performance trajectories and demonstrated a modest enhancement in catalytic activity during sustained HER operation. This performance improvement can be attributed to electrochemically induced structural reorganization of the electrodeposited NiMo framework under applied cathodic bias, leading to progressive optimization of active site accessibility and enhanced catalytic efficiency [50]. In contrast, the benchmark Pt/C catalyst underwent substantial performance degradation under identical testing conditions, underscoring the superior operational stability of the NiMo‐based systems. Remarkably, quantitative Faradaic efficiency measurement (Figure S7) reveals that the Zn‐NiMo/NF catalyst achieves 99.8% of Faradaic efficiency toward hydrogen evolution during a 20 h electrolysis period, unequivocally confirming its exceptional catalytic selectivity and operational robustness under prolonged electrochemical stress.

**FIGURE 4:**
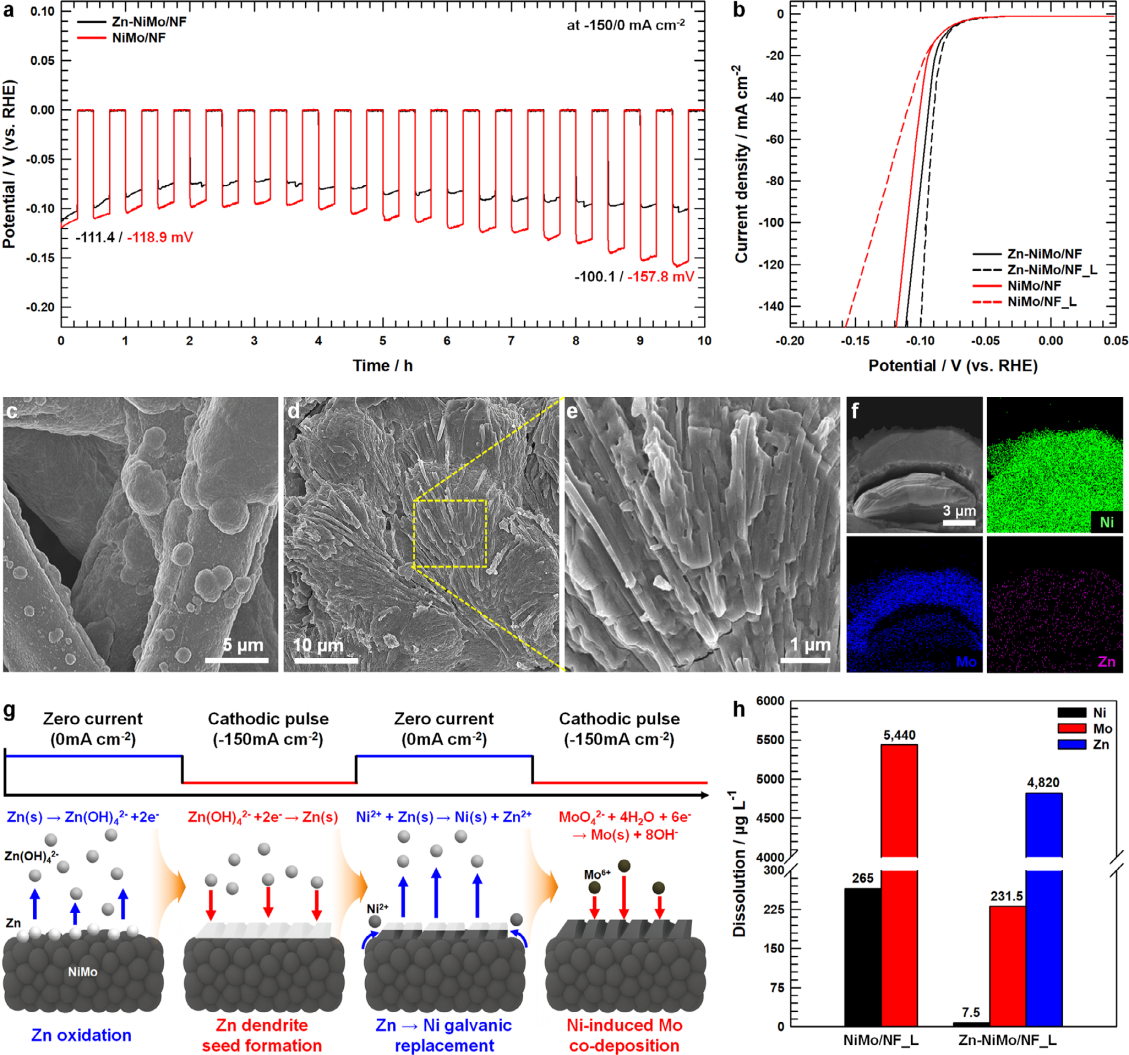
a) Load fluctuation (−150/0 mA cm^−2^) tests of Zn‐NiMo/NFand NiMo/NF for 10 h. b) LSV curves of Zn‐NiMo/NF and NiMo/NF before and after load fluctuation. FE‐SEM images of c) NiMo/NF_L and d) Zn‐NiMo/NF_L at low magnification. e) FE‐SEM image of Zn‐NiMo/NF_L at high magnification. f) SEM image at edge view with corresponding EDS elemental mapping images of Ni, Mo and Zn elements of Zn‐NiMo/NF_L electrode. g) Schematic illustration of growth of dendrites on the NiMo/NF surface in the sample during load fluctuation. h) Comparison of the metal dissolution amount from NiMo/NF_L and Zn‐NiMo/NF_L.

The inherent intermittency of renewable energy sources introduces significant operational challenges for water electrolysis systems, as fluctuating power inputs can compromise electrochemical stability. Therefore, catalyst durability under dynamic load cycling is critical for viable integration into renewable‐powered hydrogen generation infrastructure. To evaluate the resilience of NiMo/NF and Zn‐NiMo/NF catalysts under fluctuating operational conditions, chronopotentiometric measurements were performed by cyclically alternating the applied current density between −150 and 0 mA cm^−2^ at 15 min intervals over a cumulative 10 h testing period (Figure 4a). Both catalysts initially exhibited a modest activity enhancement during the early stages of load cycling, consistent with the performance trends observed during chronopotentiometric testing at −50 mA cm^−2^ (Figure 3e). However, distinct behavioral differences emerged during prolonged intermittent operation. The NiMo/NF catalyst demonstrated a progressive cathodic potential drift from −118.9 to −157.8 mV over the course of the experiment, signifying gradual performance degradation. In contrast, the Zn‐NiMo/NF catalyst exhibited substantially attenuated performance deterioration, maintaining superior electrochemical stability throughout the dynamic loading protocol. Post‐cycling LSV analysis (Figure 4b) provides further corroboration of these divergent stability profiles. The Zn‐NiMo/NF catalyst following load fluctuation (designated as Zn‐NiMo/NF_L) demonstrated marginally enhanced HER activity relative to its pristine state, requiring an overpotential of only 90.5 mV to achieve 50 mA cm^−2^. This performance improvement is fully consistent with the electrochemically induced structural reorganization and active site optimization phenomena observed during sustained cathodic polarization (Figure 3e). Conversely, the NiMo/NF catalyst after load fluctuation (NiMo/NF_L) exhibited pronounced activity degradation, necessitating an increased overpotential of 112 mV at 50 mA cm^−2^—a substantial deviation from its initial performance characteristics. These comparative findings unequivocally demonstrate that zinc incorporation confers critical stabilization benefits under dynamic operational regimes, effectively mitigating the performance degradation mechanisms that compromise unmodified NiMo catalysts during intermittent electrolysis operation. FE‐SEM analysis conducted following the dynamic load cycling protocol (Figure 4c,d) revealed pronounced morphological disparities between the two catalyst systems. The NiMo/NF_L exhibited substantial catalyst delamination from the nickel felt substrate (Figure S8), which constitutes a primary mechanism underlying the observed loss in catalytic performance. In stark contrast, Zn‐NiMo/NF_L demonstrated no discernible evidence of catalyst detachment or leaching under FE‐SEM examination, underscoring the critical role of zinc in enhancing mechanical and electrochemical stability at the catalyst‐substrate interface. Intriguingly, the surface morphology of Zn‐NiMo/NF_L underwent a dramatic transformation during dynamic cycling, evolving from the initially smooth topology of pristine Zn‐NiMo/NF into a highly ramified dendritic architecture (Figure 4e)—a morphological motif strongly reminiscent of zinc dendrite formation commonly encountered in rechargeable zinc‐based electrochemical energy storage systems [40, 51]. This dendritic restructuring contributed to a measurable enhancement in the electrochemically accessible surface area of Zn‐NiMo/NF following load fluctuation testing (Figure S9). To elucidate the compositional nature of these dendritic protrusions and determine whether they comprise exclusively zinc or represent a co‐deposited Zn─Ni─Mo composite phase, edge‐view SEM analysis was performed. The Zn‐NiMo/NF_L (Figure 4f) reveals a structurally intact underlying NiMo layer surmounted by a distinctly delineated dendritic overlayer enriched in NiMo constituents, suggesting a complex multi‐element growth mechanism rather than simple zinc redeposition. To examine whether the alloy characteristics of the basal NiMo layer are reasonably preserved within the reconstructed surface, an edge‐view SEM‐EDS line‐scanning analysis (Figure S10) was conducted to compare the underlying alloy with the surface‐generated NiMo‐rich dendritic sites. The basal NiMo layer exhibited a Ni:Mo ratio of 6.63:1.0, whereas the dendritic region showed a ratio of 6.47:1.0. This observation suggests that the alloy character of the catalyst remains largely unchanged during reconstruction, providing a reasonable basis for the preservation of intrinsic catalytic performance.

Under dynamic load cycling conditions (Figure 4g), the electrodeposited zinc layer undergoes oscillatory growth‐dissolution transitions, forming dendritic protrusions during cathodic polarization phases while experiencing preferential oxidative stripping under open‐circuit relaxation [52]. This sacrificial electrochemical behavior continuously sequesters parasitic oxidation processes and attenuates interfacial potential excursions at the catalyst‐electrolyte boundary, thereby shielding the underlying active phase from degradation. Concurrently, trace Ni^2+^ ions released through partial dissolution of the parent NiMo catalyst undergo spontaneous galvanic displacement reduction on the transiently formed zinc structures. Given the substantial electrochemical potential difference between the Ni^2+^/Ni (−0.25 V vs SHE) and Zn^2+^/Zn (−0.76 V vs SHE) redox couples, thermodynamic considerations favor spontaneous nickel reduction on the zinc surface, generating a conformal nickel‐enriched passivation shell [53, 54]. During subsequent cathodic polarization cycles, molybdenum species that were initially dissolved during anodic or load‐fluctuating conditions can be re‐incorporated onto nickel‐rich surfaces through an electrochemically induced co‐deposition mechanism [55]. In this process, metallic nickel facilitates the reduction of molybdenum ions. The renewed incorporation of molybdenum progressively transforms the nickel‐rich layer into hierarchical NiMo‐enriched dendritic frameworks that exhibit enhanced electronic conductivity and a substantially increased density of catalytically active sites. Notably, whereas conventional cyclic charging–discharging protocols in electrochemical systems typically promote tip‐directed, vertically oriented dendritic growth driven by heterogeneous nucleation and localized ionic supersaturation, the dendritic overlayer observed in the present system follows a fundamentally distinct morphological evolution pathway [30, 31]. Rather than propagating vertically toward the counter electrode—as commonly observed in zinc‐based battery architectures—these dendrites exhibit pronounced lateral extension along the catalyst surface plane. Across all four representative SEM images, including the high magnification domain spots 1–3 (Figures S11–S13), and the low magnification spot 4 (Figure S14), the corresponding orientation histograms consistently exhibit pronounced anisotropic peaks rather than angle‐independent isotropic distributions. Such behavior is fundamentally incompatible with tip‐like vertical dendrite growth, which typically generates isotropic angular distributions due to 3D branching and protrusion. Instead, the persistence of a dominant orientation peak across all sampled SEM regions provides quantitative evidence that the reconstructed dendrites predominantly extend laterally through lamellar spreading along the surface rather than vertically outward. The lateral growth orientation can be explained by the coupled effects of interfacial electric‐field distribution, mass transport, and kinetic moderation at the NiMo surface. The gently curved topography generates spatial variations in field intensity, causing locally enhanced tangential current densities that favor lateral ion‐reduction pathways over normal deposition [56, 57]. Simultaneously, diffusion limitations exacerbated by bubble formation via HER hinder ion transport perpendicular to the surface, generating anisotropic concentration gradients oriented parallel to the substrate [58]. These conditions promote preferential lateral ion flux and sustain sideward dendritic propagation. Furthermore, this behavior aligns with the Damköhler number framework (Da ≈ *J*_0_/*J*_lim_), which defines whether electrodeposition proceeds under reaction‐controlled or transport‐controlled conditions [58]. When Da < 1, shallow diffusion fields favor 2D nucleation and lateral extension. In our system, HER competes strongly with Zn^2+^ → Zn^0^ reduction, moderating the effective deposition rate and shifting the interface toward this transport‐balanced regime [59, 60]. As a result, steep concentration gradients necessary for vertical tip amplification do not develop. Additionally, the smooth NiMo/NF surface minimizes electric‐field focusing and suppresses geometrically driven perturbations that typically initiate vertical dendrite growth [61]. Consequently, the initially deposited Zn adopts a lateral type morphology, which subsequently templates the laterally oriented NiMo‐rich dendritic passivation layer observed after cycling.

Critically, unlike vertically propagating dendrites in battery systems—which pose substantial risks of internal short‐circuiting and catastrophic cell failure—these laterally oriented structures form a conformal protective overlayer intimately integrated with the underlying alloy surface. Far from being deleterious, this architectural motif appears to function as a robust structural scaffold that effectively suppresses active site dissolution and contributes to both enhanced electrochemically accessible surface area and superior durability under intermittent load cycling conditions. Furthermore, the dendritic surface generated after reconstruction is expected to confer additional advantages during HER operation. Metallic dendritic structures are known to exhibit favorable capillary behavior that promotes rapid liquid replenishment, shortens bubble residence time, and facilitates efficient disruption of the liquid gas interface through capillary driven forces [62]. These effects substantially enhance bubble detachment and mitigate bubble induced concentration gradients, thereby reducing physical dissolution issue and local mass transport resistance. As shown in Figure S15, the Zn‐NiMo/NF_L electrode maintains slightly higher activity than Zn‐NiMo/NF even after ECSA normalization. In addition, the reconstructed catalyst exhibits a Tafel slope of 29.6 mV dec^−1^, which is slightly lower than that measured prior to reconstruction. These observations suggest that the activity enhancement after surface reconstruction can be attributed to a combination of electrochemically induced surface reorganization under applied potential and secondary effects associated with the laterally oriented dendritic morphology, including improved bubble removal and reduced mass transport resistance. Moreover, the dendritic NiMo overlayer may attenuate substrate dissolution through electrochemical current redistribution, wherein the protruding dendritic features preferentially draw cathodic current away from the underlying NiMo base layer, thereby reducing the effective current density experienced by the substrate [63]. This current‐shielding effect mitigates electrochemical stress on the base catalyst, suppressing dissolution‐driven degradation pathways. During zero‐current relaxation intervals, the partially occluding dendritic overlayer introduces substantial increases in interfacial tortuosity and diffusion boundary‐layer thickness, effectively reducing the apparent diffusivity of electrolyte species and elongating mass‐transport pathways to the underlying NiMo surface [64]. This mass‐transfer impedance attenuates the flux of oxidizing species to the base layer, thereby retarding nickel and molybdenum oxidation as well as potential dealloying processes at the substrate interface, while simultaneously preserving catalytic activity at the electrochemically accessible dendritic surfaces exposed to the electrolyte. Additionally, the sacrificial oxidation propensity of zinc during dendritic formation, coupled with the presence of residual zinc within the composite structure, likely contributes synergistically to suppressing dissolution of the NiMo‐enriched dendritic layer itself. This multifaceted protective mechanism—encompassing geometric current redistribution, mass‐transport modulation, and sacrificial redox buffering—collectively accounts for the exceptional operational stability exhibited by the Zn‐NiMo/NF catalyst under dynamic loading protocols, representing a sophisticated self‐stabilizing electrochemical architecture uniquely suited for intermittent renewable energy‐driven electrolysis applications.

To rigorously assess whether the dendritic passivation layer effectively inhibits active site dissolution and to evaluate potential electronic structure modifications induced by dynamic load cycling, comprehensive XPS analysis was performed on both NiMo/NF_L and Zn‐NiMo/NF_L (Figures S16 and S17). Following prolonged cycling, the NiMo/NF exhibited a pronounced intensification of the metallic Ni^0^ photoelectron signal within the Ni 2p_3/2_ spectral envelope, accompanied by the emergence of a distinct Ni° feature at 869 eV in the Ni 2p_1/2_ region—spectroscopic signatures diagnostic of exposed metallic nickel felt substrate resulting from substantial catalyst delamination. Furthermore, the appearance of additional satellite features (designated as satellite B) following load fluctuation, while the predominant oxidation state remains Ni^2+^, can be attributed to enhanced shake‐up processes and multiplet splitting arising from ligand‐field perturbations induced by repetitive electrochemical cycling [65]. In contrast, the Zn‐NiMo/NF retained minimal Ni^0^ signal intensity even after exhaustive load fluctuation testing, unambiguously confirming that the dendritic overlayer functions as an effective diffusion barrier that physically impedes dissolution of the underlying NiMo active phase. Notably, the Ni 2p satellite structure remained singularly well‐defined without additional splitting, further corroborating the exceptional structural and chemical resilience of this catalyst architecture under dynamic current modulation. Complementary XPS analysis of the O 1s spectral region (Figure S17) reveals a striking disparity in the M─OH to M─O intensity ratio between NiMo/NF_L (0.075) and Zn‐NiMo/NF_L (0.037). The substantially elevated hydroxide fraction observed for NiMo/NF_L appears intrinsically linked to ligand‐field modifications correlated with the emergence of the satellite B feature, suggesting progressive surface hydroxylation accompanying structural degradation. To comprehensively elucidate elemental dissolution kinetics under dynamic loading conditions, post‐electrolysis electrolyte solutions were subjected to inductively coupled plasma mass spectrometry (ICP‐MS) analysis. As shown in Figure 4h and Table S3, the Zn‐NiMo/NF_L exhibited predominantly zinc leaching (4,820 µg L^−1^), while demonstrating remarkably suppressed nickel and molybdenum dissolution (7.5 and 231.5 µg L^−1^, respectively). Conversely, the NiMo/NF_L displayed substantially elevated nickel and molybdenum leaching (265 and 5,440 µg L^−1^, respectively), representing increases of more than one order of magnitude. Given the well‐established identification of nickel as the principal catalytically active element for hydrogen evolution in NiMo‐based electrocatalysts [10, 11], these quantitative dissolution data provide compelling evidence that the dendritic passivation architecture plays an indispensable role in preserving the integrity of nickel active sites by effectively mitigating their electrochemical dissolution under fluctuating operational conditions. Raman spectroscopy performed after the load fluctuation test (Figure S18) revealed a distinct Ni─O stretching mode peak at 543 cm^−1^ [37] for NiMo/NF_L, indicating the irreversible nickel oxidation under the fluctuating current conditions. In contrast, Zn‐NiMo/NF_L retained its metallic characteristics, showing no detectable oxide‐related Raman features, consistent with its pristine state. These results confirm that while NiMo/NF underwent surface oxidation during repeated load variations, the presence of the Zn in Zn‐NiMo/NF effectively suppressed such oxidative degradation.

**FIGURE 5:**
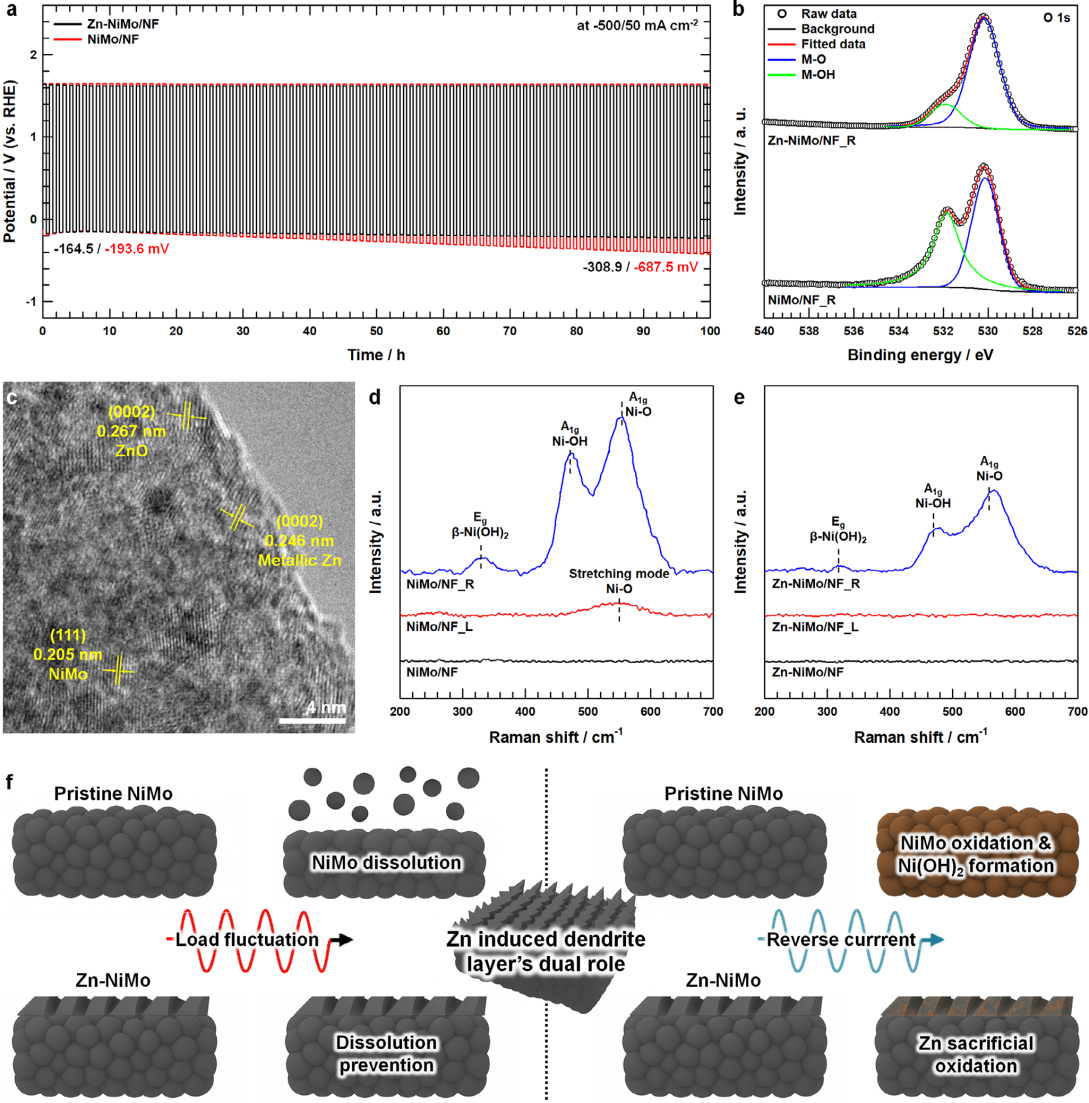
a) Load fluctuation tests (−500/50 mA cm^−2^) of Zn‐NiMo/NF and NiMo/NF for 100 h. b) XPS O 1s spectra of Zn‐NiMo/NF_R and NiMo/NF_R. c) HR‐TEM image of Zn‐NiMo/NF_R. Raman spectra (200–700 cm^−1^) of d) NiMo/NF, NiMo/NF_L, NiMo/NF_R and e) Zn‐NiMo/NF, Zn‐NiMo/NF_L, Zn‐NiMo/NF_R. f) Schematic illustration of the dual role of the dendrite layer in preventing NiMo dissolution under load fluctuation and undergoing sacrificial oxidation under reverse current.

To accurately simulate the reverse current transients frequently encountered during intermittent operation of renewable energy‐powered electrolyzers and to rigorously substantiate the sacrificial oxidation behavior of zinc, a more stringent accelerated degradation protocol was implemented. Specifically, the applied current density was cyclically alternated between −500 mA cm^−2^ (cathodic) and +50 mA cm^−2^ (anodic) at 30 min intervals over a 100 h duration, thereby imposing both reductive and oxidative electrochemical stresses on the catalyst surface. As shown in Figure 5a, under these substantially harsher operating conditions, the NiMo/NF exhibited pronounced performance degradation, with the cathodic overpotential progressively deteriorating from −193.6 mV to −687.5 mV—representing a far more severe decline compared to the moderate −150/0 mA cm^−2^ cycling protocol (Figure 4a). In stark contrast, the Zn‐NiMo/NF demonstrated markedly attenuated potential drift throughout the test duration (−164.5 to −308.9 mV), unambiguously highlighting the protective functionality of the zinc‐mediated dendritic overlayer through synergistic mechanisms encompassing both physical passivation and sacrificial electrochemical buffering. ICP‐MS results (Figure S19 and Table S4) reveals that dissolution of catalytically active species from Zn‐NiMo/NF following reverse current‐inclusive load fluctuation (designated Zn‐NiMo/NF_R) was substantially suppressed relative to NiMo/NF subjected to identical testing conditions (NiMo/NF_R). Furthermore, post‐cycling polarization measurements (Figure S20) demonstrate that Zn‐NiMo/NF_R retained considerable catalytic performance with an overpotential of 116.8 mV at 50 mA cm^−2^, whereas NiMo/NF_R exhibited pronounced activity loss, requiring 165.7 mV at equivalent current density. The modest performance attenuation observed for Zn‐NiMo/NF_R can be attributed to unavoidable surface oxidation occurring during the anodic reverse‐current intervals. Nevertheless, the magnitude of this degradation was substantially mitigated compared to NiMo/NF_R, strongly suggesting that zinc incorporation provides effective protection against oxidative surface passivation even under aggressive bidirectional current cycling. Post‐mortem XRD analysis following the accelerated degradation protocol (Figure S21) provided compelling crystallographic evidence for divergent structural stability. The diffraction pattern of NiMo/NF_R exhibited complete disappearance of the characteristic shoulder peak corresponding to the NiMo alloy phase—a structural signature unambiguously attributable to extensive electrochemical dissolution of the NiMo framework under severe cycling conditions [66]. Conversely, the Zn‐NiMo/NF_R catalyst retained this diagnostic shoulder feature with minimal intensity loss, definitively confirming that the sacrificial dendritic overlayer effectively shielded the underlying NiMo structure from dissolution‐driven degradation.

XPS analysis conducted following the reverse current‐simulated load fluctuation protocol (Figure S22) reveals substantive alterations in the surface chemical environment of nickel. The NiMo/NF_R exhibited markedly intensified satellite B features relative to NiMo/NF_L (Figure S16), indicating that the periodic anodic current intervals induced progressive transformation of surface nickel species—a phenomenon attributable to the structural and chemical vulnerability of NiMo alloys under oscillatory electrochemical stress. Conversely, the Zn‐NiMo/NF_R maintained comparatively weak metallic Ni^0^ and satellite B signatures even after experiencing multiple anodic polarization cycles, demonstrating superior resistance to surface oxidation. Furthermore, high‐resolution O 1s XPS spectra (Figure 5b) reveal a substantially elevated M─OH/M─O intensity ratio for NiMo/NF_R (0.979) compared to Zn‐NiMo/NF_R (0.188). This pronounced disparity unambiguously confirms that zinc incorporation and the resultant dendritic passivation architecture effectively suppress nickel oxidation and hydroxylation processes, as well as alloy dissolution, thereby preserving the integrity of the catalytically active NiMo surface throughout dynamic load cycling. These findings are further supported by the HR‐TEM and Raman spectroscopy results obtained after the reverse current‐mimicked load fluctuation test. HR‐TEM examination of NiMo/NF_R (Figure S23) reveals lattice fringes with d‐spacing of 0.236 nm, assignable to the (101) crystallographic plane of β‐Ni(OH)_2_, constituting definitive evidence of irreversible surface phase transformation [67]. In contrast, Zn‐NiMo/NF_R (Figure 5c) predominantly retains the characteristic metallic NiMo lattice fringes (*d* = 0.205 nm), while also exhibiting additional lattice spacings of 0.266 nm corresponding to the (0002) plane of zinc oxide [46]. This observation provides direct structural evidence that zinc undergoes preferential sacrificial oxidation, thereby electrochemically shielding nickel from irreversible hydroxide formation. Furthermore, as shown in Figure S25a, Zn species remain distributed along the catalyst surface even after long‐term reverse‐current mimicked protocol. During galvanic perturbation, Zn dissolution does not simply remove the sacrificial layer but instead initiates a continuous sequence of precipitation, dehydration, and reorganization processes that collectively maintain a stable ZnO‐rich interfacial layer. Even when a portion of the Zn is consumed, the high local alkalinity and the rapid formation kinetics of Zn(OH)_2_ and ZnO ensure that a passivating ZnO skin is promptly regenerated [68]. Rather than behaving as a transient by‐product, this ZnO layer becomes an integral part of the evolving catalyst interface, repeatedly rebuilding itself whenever the surface experiences oxidative stress or dissolution events. Raman spectroscopy analysis (Figure 5d,e) further revealed distinct differences in surface oxidation behavior between the two catalysts under load fluctuation conditions. In the case of NiMo/NF_R, prominent vibrational modes were observed at 317 cm^−1^ (*E*_g_), 473 cm^−1^ (A_1g_) and 565 cm^−1^ (A_1g_), which are characteristic of β‐Ni(OH)_2_, Ni─OH and Ni─O lattice vibrations in nickel oxide/hydroxide species, respectively [37, 69]. In contrast, Zn‐NiMo/NF_R exhibited significantly weaker Ni─OH and Ni─O related Raman bands, demonstrating that the Zn sacrificial layer effectively suppressed Ni oxidation and Ni(OH)_2_ transition during load fluctuation, thereby preserving the structural integrity of the NiMo surface. This protective phenomenon can be mechanistically rationalized through the preferential anodic oxidation of zinc to soluble zincate species (Zn + 4OH^−^ → Zn(OH)_4_^2−^) during reverse current intervals. This electrochemical process effectively shifts the local mixed potential at adjacent nickel sites toward more cathodic values, thereby diminishing the thermodynamic driving force for nickel‐to‐nickel hydroxide conversion [70]. Additionally, zincate formation consumes hydroxide ions from the interfacial region, which further mitigates β‐Ni(OH)_2_ nucleation and accumulation at the catalyst surface—a critical degradation pathway under anodic polarization. Figure S24 presents the local pH‐time profiles obtained from the operando OCP measurements. For Zn‐NiMo/NF, the local pH increases relatively rapidly from 13.94 to about 14.32 during the first few hours of load fluctuation, and then rises more gradually to approximately 14.37, remaining saturated in this highly alkaline regime until the end of the test. In contrast, the local pH near NiMo/NF initially drops slightly below the bulk value to around 13.93, then gradually increases and eventually saturates at 13.97. The initial behavior of NiMo/NF can be rationalized by considering the surface activation that occurs during the anodic‐current segments of the load‐fluctuation protocol. During the first few cycles, metallic Ni is irreversibly oxidized to Ni(OH)_2_, which transiently immobilizes OH^−^ within a thin interfacial layer. This consumption of interfacial OH^−^ results in a small initial dip of the local pH below the bulk value. Once most accessible Ni sites have been converted to Ni(OH)_2_, further Ni oxidation is limited, and the OH^−^ generated during the −500 mA cm^−2^ segments no longer encounters a substantial additional sink. Under these conditions, the produced OH^−^ rapidly equilibrates between the interface and the bulk electrolyte, so the local pH recovers to slightly above the bulk value and then remains essentially time‐independent. In contrast, in the Zn‐NiMo system, dissolved Zn is stabilized as Zn(OH)_4_^2‐,^ which remain in dynamic equilibrium with surface ZnO. This equilibrium between Zn(OH)_4_^2–^ and ZnO acts as a self‐regulating buffer that continually exchanges OH^−^ with the surrounding electrolyte, thereby maintaining an OH^−^ rich interfacial layer. As a result, the introduction of Zn provides a persistent redox‐buffering effect that suppresses irreversible Ni oxidation and stabilizes the interfacial microenvironment. Therefore, the strategic incorporation of a zinc sacrificial overlayer, which spontaneously evolves into a 3D dendritic passivation architecture under dynamic electrochemical conditions, establishes a synergistic dual‐protection mechanism for the NiMo catalyst under realistic intermittent hydrogen evolution operating conditions (Figure 5f). This in situ reconstructed dendritic layer functions simultaneously as: (i) a physical diffusion barrier that effectively suppresses electrochemical dissolution and leaching of catalytically active NiMo constituents, thereby preserving the catalyst's surface architecture and composition; and (ii) a sacrificial redox mediator that preferentially undergoes oxidation during anodic excursions, thereby electrochemically buffering the underlying nickel phase from irreversible oxidation and hydroxylation.

**FIGURE 6:**
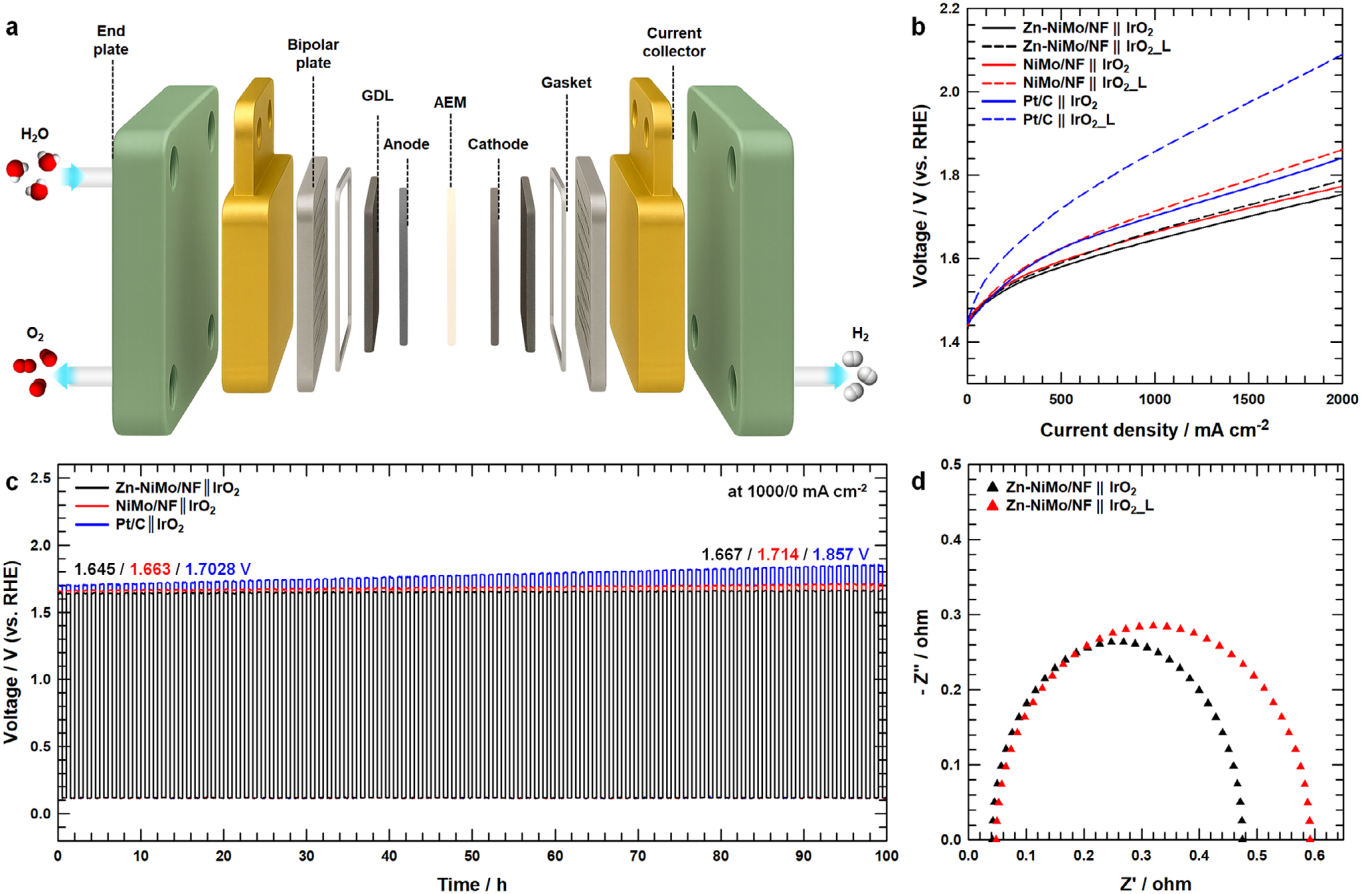
AEMWE operation of Zn‐NiMo/NF. a) schematic illustration of the AEMWE single cell and b) *I*–*V* polarization curves of Zn‐NiMo/NF || IrO_2_, NiMo/NF || IrO_2_ and Pt/C || IrO_2_ for AEMWE before and after load fluctuation. c) AEMWE performance under dynamic operation at 1000/0 mA cm^−2^ with 30 min intervals for 100 h. d) Nyquist plots of Zn‐NiMo/NF || IrO_2_ single cell before and after load fluctuation test.

To assess the practical viability of Zn‐NiMo/NF as a cathode material in operational water splitting systems and to verify that the laterally propagating dendritic architecture does not compromise long‐term durability in full‐cell configurations, an anion exchange membrane water electrolyzer (AEMWE) single cell was constructed (Figure 6a). Iridium oxide (IrO_2_) was deposited onto nickel felt via ultrasonic spray coating to serve as the oxygen evolution anode. For comparative evaluation, a commercial Pt/C benchmark catalyst was spray‐coated onto carbon paper to function as the hydrogen evolution cathode. A Sustainion X37‐50 anion exchange membrane was employed as the electrolyte separator, and all single‐cell measurements were conducted at an operating temperature of 60°C. As shown in Figure 6b, the AEMWE configuration employing Zn‐NiMo/NF as the cathode demonstrated considerable electrochemical performance, achieving a current density of 1.0 A cm^−2^ at a cell voltage of only 1.645 V—representing a notable voltage reduction compared to the benchmark Pt/C‐based cell, which required 1.7028 V to attain equivalent current density. Furthermore, comprehensive performance benchmarking (Table S5) confirms that the AEMWE incorporating Zn‐NiMo/NF exhibits metrics comparable to or exceeding those of state‐of‐the‐art electrocatalyst systems reported in recent literature, thereby establishing its competitiveness for practical hydrogen production applications. To rigorously evaluate the operational stability of the Zn‐NiMo/NF‐based AEMWE under intermittent operating conditions characteristic of renewable energy integration, a dynamic load fluctuation protocol was implemented wherein the current density was cyclically alternated between 1.0 A cm^−2^ and 0 mA cm^−2^ at 30 min intervals over a cumulative 100 h duration. In contrast to the NiMo/NF and Pt/C single‐cell systems—which exhibited progressive voltage increases from 1.663 to 1.714 V and from 1.7028 to 1.857 V, respectively, in order to sustain the target current density of 1.0 A cm^−2^, clearly indicating significant performance degradation—the Zn‐NiMo/NF‐based cell demonstrated outstanding electrochemical durability, showing only a minimal voltage drift (1.645 to 1.667 V) over the entire 100 h testing period (Figure 6c). Post‐cycling polarization analysis (Figure 6b) further corroborates these durability advantages. The Pt/C || IrO_2_ assembly following load fluctuation (designated Pt/C || IrO_2__L) suffered pronounced activity loss, requiring an elevated cell voltage of 1.857 V to achieve 1.0 A cm^−2^. Conversely, the Zn‐NiMo/NF || IrO_2_ configuration after equivalent load cycling (Zn‐NiMo/NF || IrO_2__L) exhibited substantially attenuated performance degradation, maintaining 1.0 A cm^−2^ operation at merely 1.667 V—even under testing protocols incorporating periodic anodic polarization stress. Electrochemical impedance spectroscopy analysis (Figure 6d) provides additional mechanistic insight into the exceptional stability characteristics. Nyquist plots reveal that the Zn‐NiMo/NF || IrO_2_ cell maintained remarkably low and stable charge‐transfer resistance, with values of 0.434 Ω prior to load fluctuation and only 0.544 Ω following the 100 h cycling protocol—representing minimal interfacial degradation. In addition, even after 100 h of harsh load‐fluctuation cycling with AEMWE single cell, residual Zn species were still detected on the surface of the Zn‐NiMo/NF electrode (Figure S25b), confirming the sustained nature of the Zn sacrificial oxidation behavior throughout prolonged operation. Collectively, these full‐cell validation results unequivocally demonstrate that the laterally oriented, zinc‐mediated dendritic passivation layer confers exceptional operational durability to AEMWE systems under intermittent load cycling—operational regimes directly relevant to renewable energy‐powered hydrogen production.

## 3 Conclusion

This work demonstrates an innovative strategy for enhancing the operational stability of earth‐abundant hydrogen evolution electrocatalysts under dynamic load fluctuations characteristic of renewable energy‐powered water electrolysis. The Zn‐NiMo/NF catalyst exhibits considerable HER activity (94.6 mV overpotential at 50 mA cm^−2^) comparable to Pt/C benchmarks, while demonstrating superior stability under intermittent operation including stringent reverse current conditions. Comprehensive characterization reveals that zinc incorporation enables a unique in situ reconstruction mechanism wherein the initially conformal zinc overlayer spontaneously evolves into laterally oriented, NiMo‐enriched dendritic architectures during dynamic cycling. This protective layer functions through synergistic dual‐protection: (i) serving as a physical diffusion barrier suppressing active site dissolution, with order‐of‐magnitude reductions in metal leaching; and (ii) operating as a sacrificial redox mediator wherein zinc preferentially oxidizes to zincate species, electrochemically buffering nickel from irreversible hydroxide formation. Single‐cell AEMWE validation confirms practical viability, achieving 1.0 A cm^−2^ at 1.645 V with exceptional 100 h cycling stability, outperforming degradation‐prone Pt/C systems. This sacrificial layer design paradigm establishes a generalizable approach for developing robust non‐noble metal electrocatalysts capable of sustained performance in grid‐flexible, renewable energy‐integrated hydrogen production infrastructure.

## Conflicts of Interest

The authors declare no conflicts of interest.

## Supporting information




**Supporting File**: advs73688‐sup‐0001‐SuppMat.docx.

## Data Availability

The data that support the findings of this study are available from the corresponding author upon reasonable request.
