# Supplementary material for: Laterally Oriented Dendritic Passivation via In Situ Zn Reconstruction for Stabilizing NiMo Catalysts under Dynamic Electrolysis
Source: Adv Sci (Weinh). 2026 Jan 12;13(16):e20103. doi: 10.1002/advs.202520103 (PMC13042939; doi:10.1002/advs.202520103)
Supplement: Supplementary file 1 — Supporting File: advs73688‐sup‐0001‐SuppMat.docx. [file ADVS-13-e20103-s001.docx]

**Supporting Information**

**Laterally Oriented Dendritic Passivation via In Situ Zn Reconstruction for Stabilizing NiMo Catalysts under Dynamic Electrolysis**

Taeyoung Jeong, Hyun-Jae Park and Myeongjin Kim*

T. Jeong, H-J.Park, Prof. M. Kim

School of Energy Engineering, Kyungpook National University, 80 Daehak-ro, Bukgu, Daegu 41566, Republic of Korea

E-mail: myeongjinkim@knu.ac.kr

**Methods**

**Materials.** Nickel(II) sulfate hexahydrate (NiSO_4_·6H_2_O, ACS reagent, ≥98%), sodium molybdate (Na_2_MoO_4_, ≥98%), sodium citrate tribasic dihydrate (Na_3_C_6_H_5_O_7_·2H_2_O, ACS reagent grade, 99.0%), ammonium hydroxide solution (NH_4_OH, 28-30% NH_3_ basis), zinc sulfate heptahydrate (ZnSO_4_·7H_2_O, ACS reagent, 99%), and 5 wt.% Nafion(0.038 mL, 5 wt.% in isopropanol) were purchased from Sigma-Aldrich and used without further purification. Nickel felt (NF) (thickness 300 μm, porosity 90%) was purchased from The Solution Korea. Hydrochloric acid (36.5-38.0%, NF, Multi-Compendial, GMP) was purchased from J.T. Baker. Commercial Pt/C (20 wt.% Pt on carbon) was purchased from Alfa Aesar. Commercial Iridium (Ⅳ) oxide (IrO_2_, 99%) was purchase from Aladdin Chemical Regent Co.

**Pretreatment of NF.** NF were cut to 2 cm x 2 cm and ultrasonically cleaned in acetone (20 min), followed by immersion in 3.0 M HCl (prepared by a 1:3 v/v dilution of concentrated HCl with DI water) for 10 min to remove surface oxides. The samples were then thoroughly rinsed with DI water and dried in an oven at 60 °C overnight prior to electrodeposition.

**Synthesis of NiMo/NF and Zn-NiMo/NF.** The NiMo plating bath was prepared by dissolving NiSO_4_·6H_2_O (80 g L^-1^), Na_2_MoO_4_ (50 g L^-1^), and Na_3_C_6_H_5_O_7_·2H_2_O (90 g L^-1^) in DI water under stirring until homogeneous. Subsequently, the bath pH was adjusted to 10.5 by dropwise addition of 28-30% NH_4_OH (final pH confirmed). NiMo was electrodeposited galvanostatically at 150 mA cm^-2^ for 10 min at room temperature using a ITECH IT6726V DC power supply. Electrodeposition was conducted in a two-electrode configuration using the pretreated NF as the cathode and a Pt wire as the anode. After deposition, the NiMo/NF electrodes were rinsed thoroughly with DI water and dried under vacuum at room temperature for 8 h.

To prepare Zn-NiMo/NF, a 0.1 M ZnSO_4_·7H_2_O aqueous electrolyte (28.76 g L^-1^) was employed. The NiMo/NF electrode was immersed in the solution and biased at a cell voltage of 1.0 V for 5 min in a two-electrode configuration with a Pt counter. The resulting Zn-NiMo/NF electrodes were rinsed with DI water and dried under vacuum at room temperature for 8 h before electrochemical testing. The total catalyst loading mass was measured to be 15.4 mg cm^-2^, while the Zn-NiMo/NF electrode exhibited a loading of 16.0 mg cm^-2^.

**Preparation of Pt/C working electrode.** Glassy carbon working electrode was prepared using a commercial 20 wt.% Pt/C catalyst. The catalyst ink was prepared by dispersing 5 mg of Pt/C in a mixed solvent containing 0.04 mL of 5 wt.% Nafion solution in isopropanol together with 1.06 mL ethanol and 0.10 mL deionized water, followed by ultrasonication for 30 min. An aliquot of 11 µL of the ink was drop cast onto a glassy carbon electrode (PINE, diameter 5 mm, geometric area 0.196 cm^2^) and the coated electrode was dried at room temperature. Under these conditions, the nominal loading of Pt/C was 0.615 mg cm^-2^, corresponding to a Pt loading of 0.123 mg_Pt_ cm^-2^.

**Electrochemical measurements : half-cell.** All electrochemical tests were performed in a standard three-electrode configuration controlled by a CHI-760E potentiostat/galvanostat. The electrochemical measurements were conducted in 1.0 M KOH aqueous electrolyte, using a catalyst-coated NF as the working electrode, an Ag/AgCl (KCl Sat.) as the reference electrode, and a Pt wire as the counter electrode. The Ag/AgCl reference was converted to the reversible hydrogen electrode (RHE) scale in 1.0 M KOH at room temperature. For the calibration, the electrolyte was saturated with ultrahigh-purity H_2_ (99.999%) for 30 min, and cyclic voltammetry (CV) were recorded at 1 mV s^-1^ using a Pt working electrode. The RHE potential was taken as the midpoint of the forward/reverse zero-current crossings. Before evaluating the HER activity of the prepared catalysts, the electrolyte was purged with ultrahigh-purity H_2_ (99.999%) for 30 min. Reported potentials were corrected for uncompensated solution resistance according to

$E_{iR-corrected} = E-iR$ (1)

where $R$ was obtained from high-frequency impedance. Electrochemical impedance spectroscopy (EIS) was collected with a 10 mV perturbation over 100 kHz-0.1 Hz.

The electrochemical surface area (ECSA) of the electrode was calculated using the equation

ECSA = C_dl_/C_s_ (2)

where Cs = 0.04 mF cm^-2^ represents the smooth plane capacitance of the electrode at 1.0M KOH.

Load fluctuation durability was examined under galvanostatic chronopotentiometry in the same cell and electrolyte. The current density, j (normalized to the geometric area), followed a square-wave profile with fixed holds at each level. Load-fluctuation durability was assessed in two separate measurements using identically prepared electrodes. In the first protocol, the electrode was cycled between -150 and 0 mA cm^-2^ with 15-min intervals at each current level to evaluate performance retention under load fluctuations. In the second protocol, the electrode was cycled between -500 and 50 mA cm^-2^ with 30-min intervals at each level to impose anodic polarization capable of inducing oxidative degradation. Results from the two protocols are reported independently. The -150/0 mA cm^-2^ and -500/50 mA cm^-2^ segments were run for total durations of 10 h and 100 h, respectively. Unless otherwise noted, all currents are reported as geometric current densities.

To confirm that the cathodic current was due to hydrogen evolution and to determine the Faradaic efficiency (FE), chronopotentiometric electrolysis was carried out at 50 mA cm^-2^ for 30 h in H_2_-saturated 1.0 M KOH. Evolved H_2_ was quantified by gas chromatography equipped with a thermal conductivity detector (Shimadzu GC-8A for Ar carrier) from headspace samples collected every 0.5 h. The theoretical H_2_ amount was computed from the integrated charge, and FE was calculated using the following equation:

$\varepsilon= \frac{n_{H_{2},meas}}{Q/(2F)} \times100$ (3)

where $n_{H_{2},meas}$ is the moles of H_2_ measured by GC, $Q$ is the total charge, and $F$ is Faraday’s constant. The measured-vs-theoretical H_2_ plots were nearly unity (**Figure S10**).

**Electrochemical measurements : single cell.** The single cell test was conducted using WPG100s potentionstat/galvanostat (WonATech, Korea). A IrO_2_ catalyst (2.0 mg cm^−2^) was ultrasonically sprayed onto 2 cm × 2 cm nickel felt as the anode. A 2 cm × 2 cm Zn-NiMo/NF was used as the cathode, and the Sustainion X37-50 was used as the anion exchange membrane. For the comparison, a Pt/C catalyst (2.0 mg cm^−2^) was ultrasonically sprayed onto 2 cm × 2 cm carbon paper (0.2 mm-thick, The solution, Korea) as the cathode. An air compressor was utilized to apply 8 bar pressure and secure the test electrodes, while the hydraulic single cell was maintained at 60 °C by circulating water. The electrolyte (1 M KOH) was delivered from the anode side at a flow rate of 4 mL min^-1^ using a peristaltic pump. Polarization curves were recorded in the voltage range of 1.3-2.2 V, and EIS was carried out at 200 mA cm^-2^ over a frequency window of 10^5^ to 1 Hz. The load-fluctuation durability test was conducted by alternating the current density between 1000 and 0 mA cm^-2^, with 30 min holds at each level, over a total period of 100 h.

**Characterization.** The morphologies of the samples were analyzed using field-emission scanning electron microscopy (FE-SEM, Hitachi SU8220) and high-resolution transmission electron microscopy (HR-TEM, Titan G2 ChemiSTEM Cs Probe) at 200 kV. Field-emission transmission electron microscopy (FE-TEM) was performed on a Titan G2 ChemiSTEM Cs Probe operated at 200 kV. High-angle annular dark-field scanning transmission electron microscopy (HAADF-STEM) and the corresponding energy-dispersive X-ray spectroscopy (EDS) elemental mapping were conducted on a probe-corrected Titan G2 ChemiSTEM Cs Probe (200 kV) equipped with a spherical-aberration corrector and an EDS detector. Elemental analyses were conducted on FlashSmar^TM^ Elemental Analyzer in CHN Mode. X-ray diffraction (XRD, New D8-Advance, Bruker-AXS) patterns were collected at a scan rate of 1 s^-1^ within the 2θ range of 10^o^-80^o^ and using Cu K_α1_ radiation (0.154056 nm). X-ray photoelectron spectroscopy (XPS, ThermoFisher NEXSA) analysis was performed with a monochromatic Al Kα (hν = 1486.6 eV) X-ray source. [Raman spectra were recorded using a Raman spectrometer (inVia Reflex, Renishaw)](https://www.sciencedirect.com/topics/chemistry/raman-spectrum) equipped with a 532 nm excitation laser. Directionality histograms were extracted using the Fourier transform-based algorithm implemented in Fiji (ImageJ). The local pH values were obtained using the Nernst equation. In alkaline media, the HER can be expressed as H_2_ + 2OH^-^ ↔ 2H_2_O + 2e^-^. Applying the Nernst equation to this reaction gives an electrode potential of the form :

E = E_0_ + (RT/F) ln a(OH^-^) (4)

Using the relationship between pH and pOH, this expression can be rearranged to :

E - E_RHE_ ≈ 0.059(pH - 14) (5)

at room temperature, where 2.303 RT/F is approximately 0.059 V per pH unit. The potential difference between the interface and the bulk solution, ΔE = E_local_ - E_bulk_, can therefore be written as ΔE ≈ 0.059(pH_local_ - pH_bulk_), leading to :

pH_local_ = pH_bulk_ + ΔE/0.059^[1]^ (6)

In our analysis, pH_bulk_ was measured to be 13.94 using a calibrated pH meter.


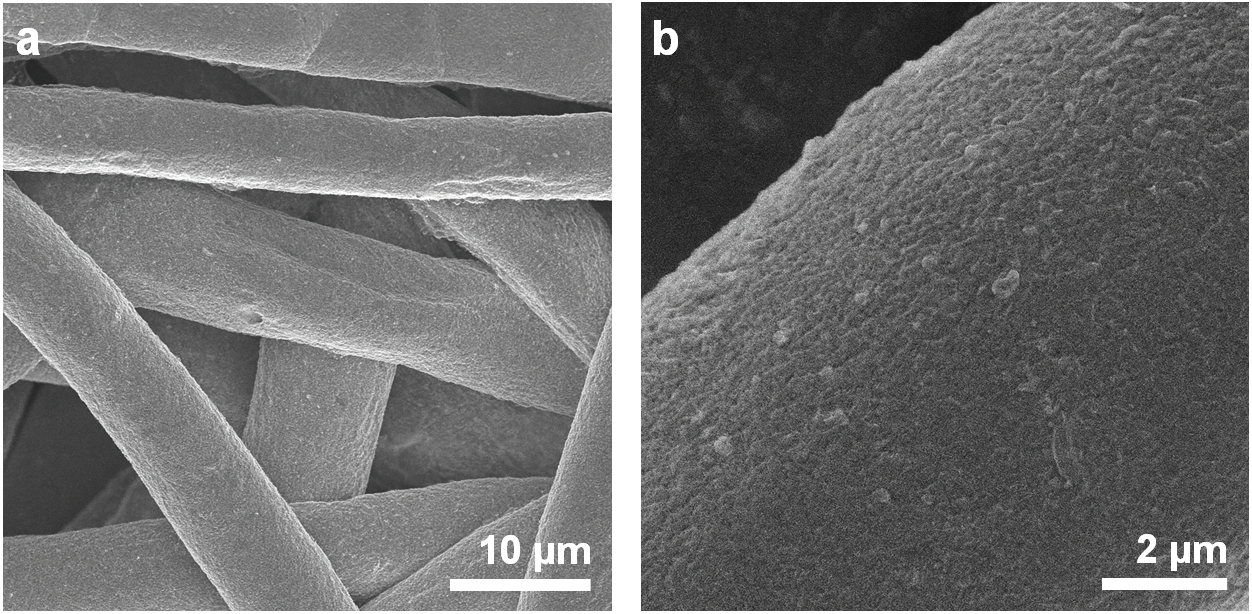


**Figure S1.** Field emission scanning electron microscopy (FE-SEM) images of nickel felt (NF) at (a) low magnification and (b) high magnification.


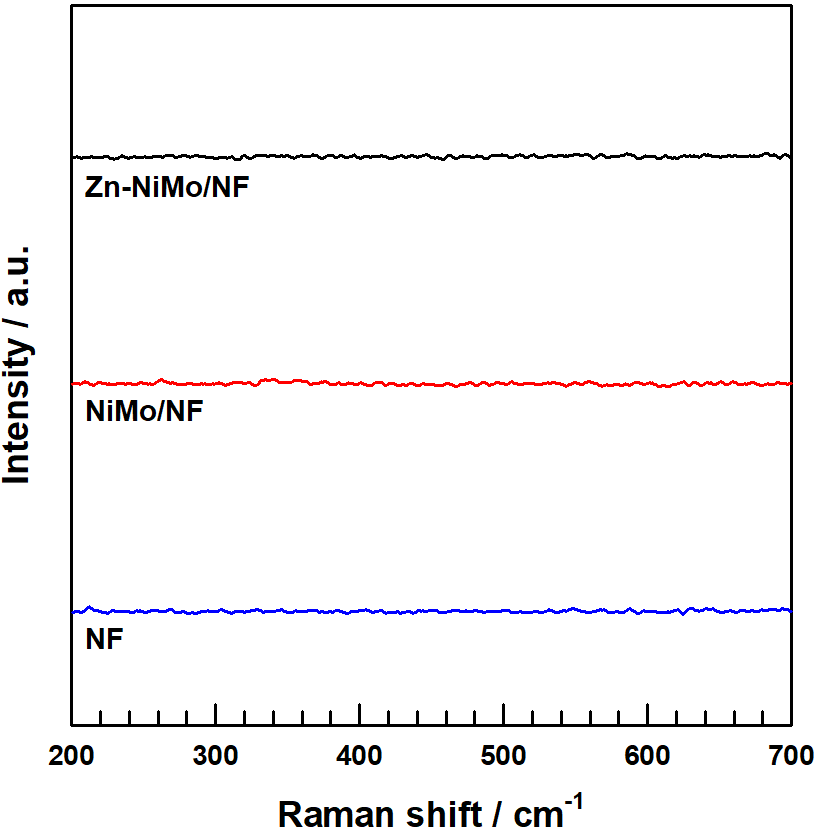


**Figure S2.** Raman spectra of Zn-NiMo/NF, NiMo/NF and NF in the range of 200-700 cm^-1^.


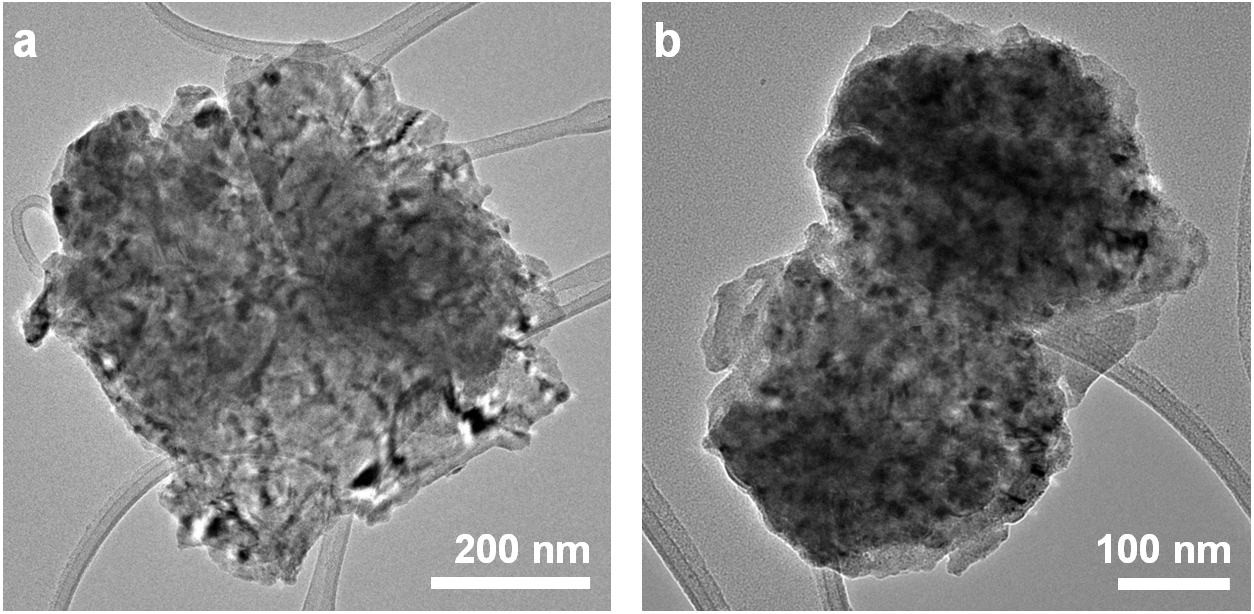


**Figure S3.** High-resolution transmission electron microscopy (HR-TEM) image of (a) NiMo/NF and (b) Zn-NiMo/NF.


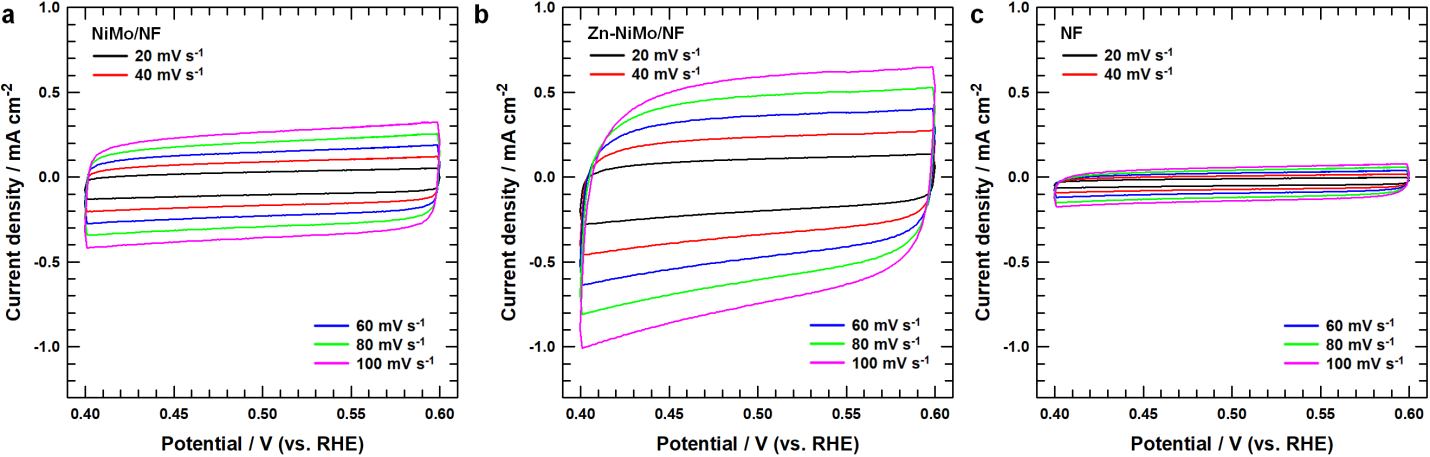


**Figure S4.** Cyclic voltammetry (CV) scans of (a) NiMo/NF, (b) Zn-NiMo/NF, and (c) Ni felt in a non-faradaic current region (0.4-0.6 V vs. RHE) at different scan rates of 20, 40, 60, 80, and 100 mV s^-1^.


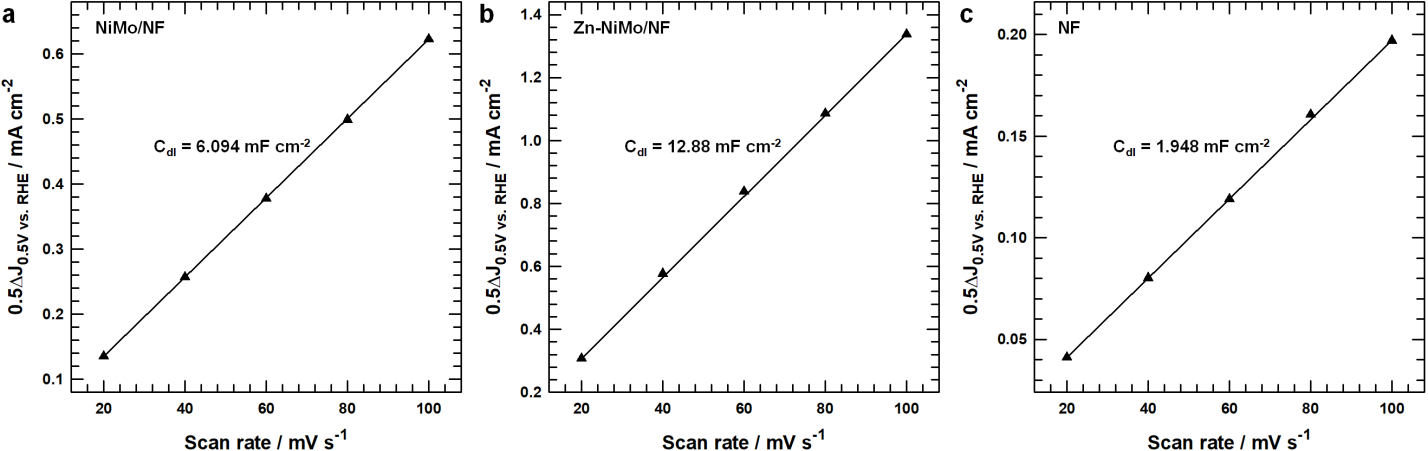


**Figure S5.** Linear fitting of the capacitive current as a function of CV scan rate for (a) NiMo/NF, (b) Zn-NiMo/NF, and (c) NF.


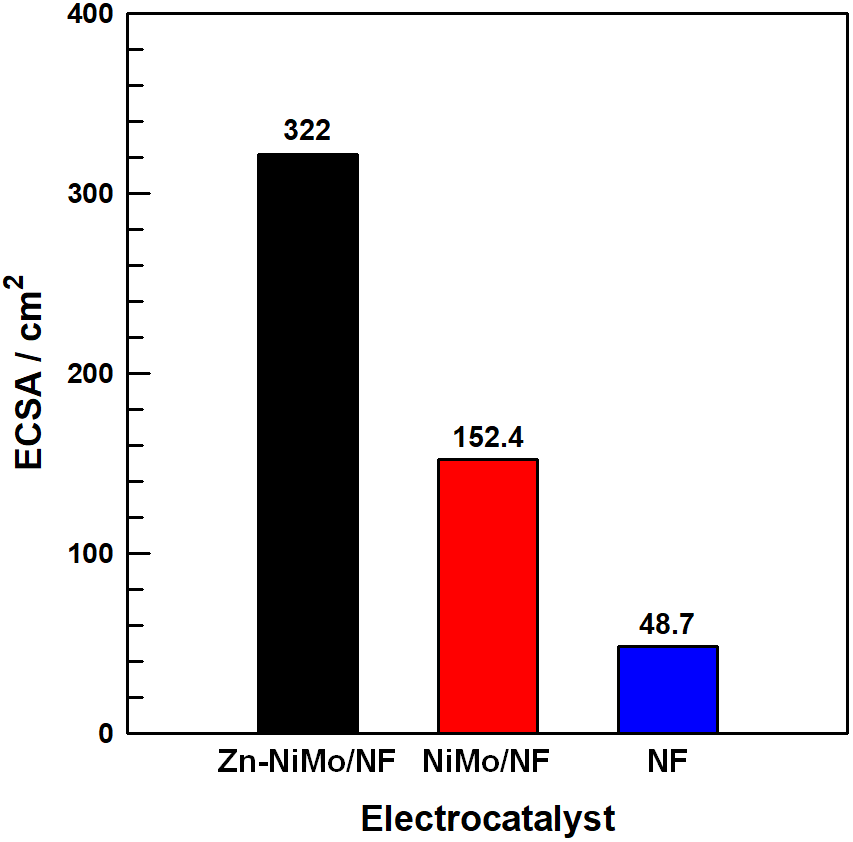


**Figure S6.** Comparison of the electrochemical surface area (ECSA) for Zn-NiMo/NF, NiMo/NF, and NF.


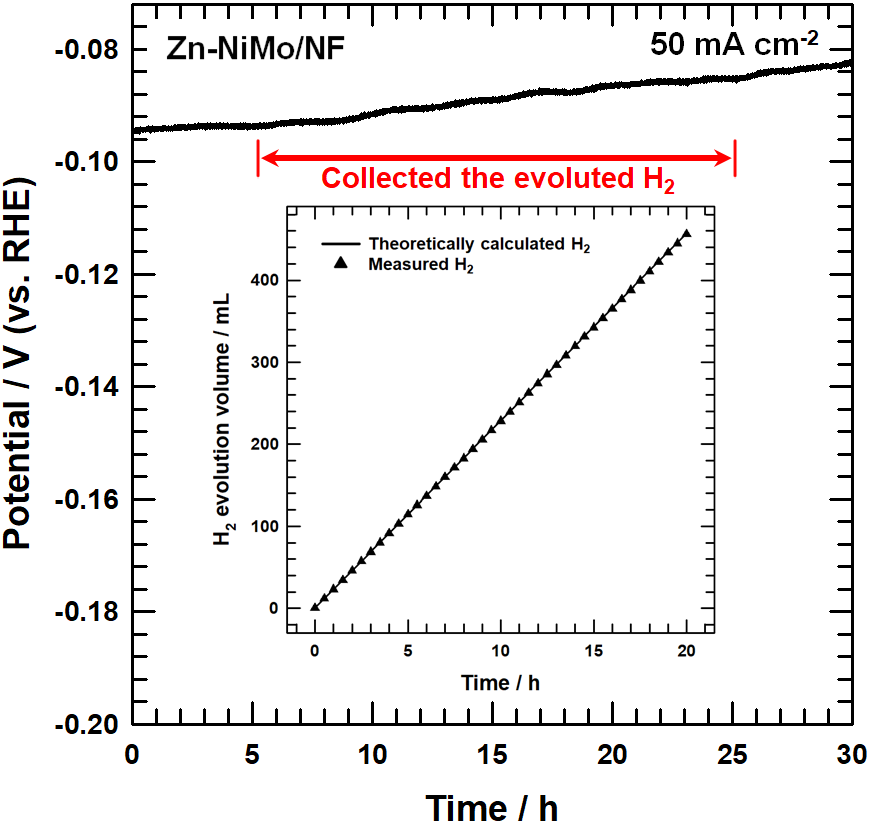


**Figure S7.** Theoretical and experimental H_2_ gas evolution volume of Zn-NiMo/NF for 20 h during chronopotentiometry (CP) measurement at a constant current density of 50 mA cm^-2^.


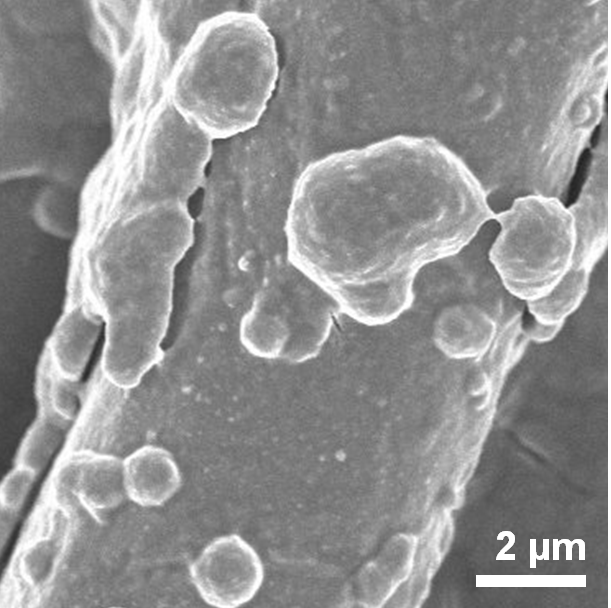


**Figure S8.** FE-SEM image of the dissolution site on NiMo/NF_L at high magnification.


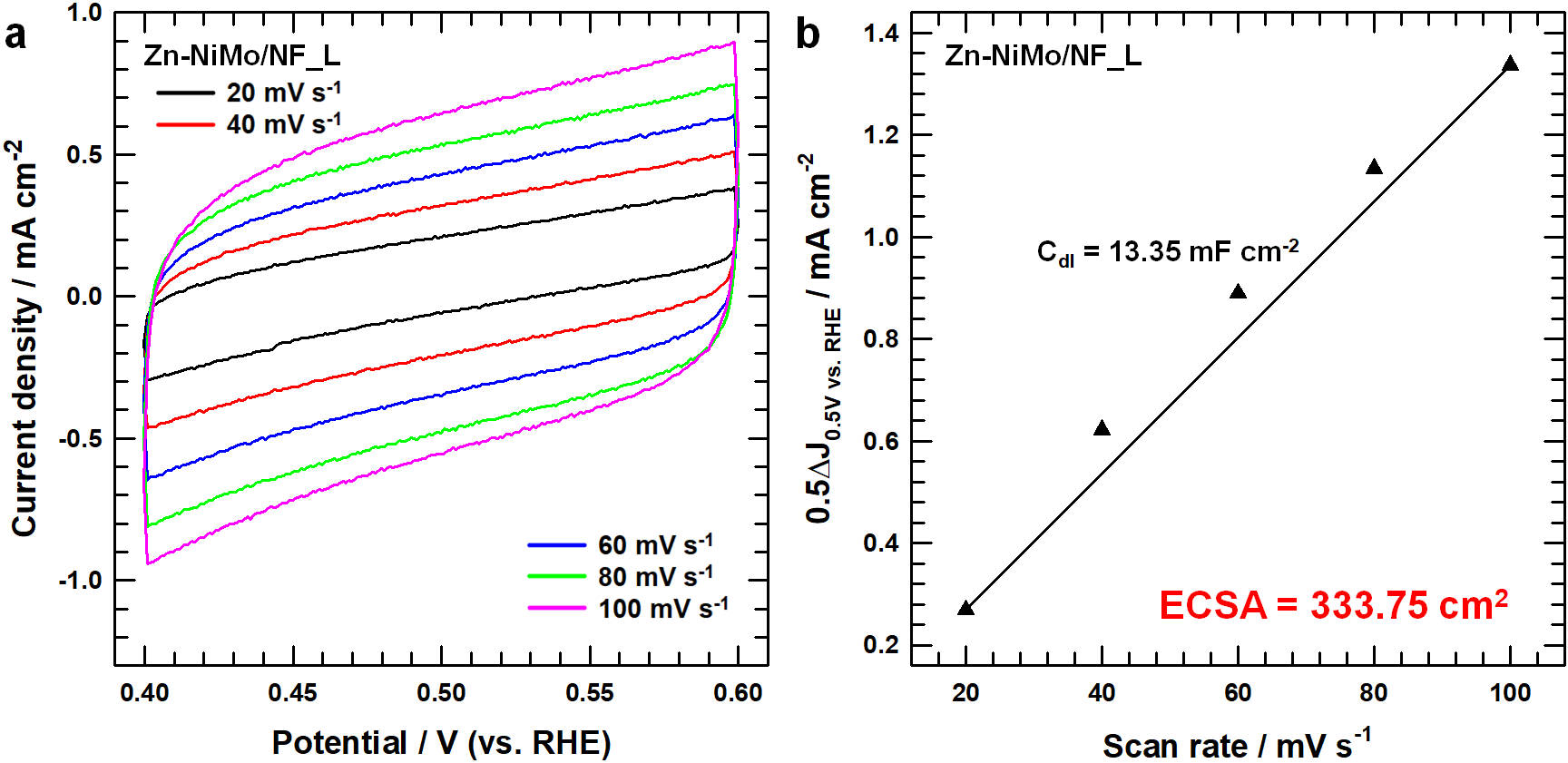


**Figure S9.** (a) CV scans of Zn-NiMo/NF_L in a non-faradaic potential region (0.40-0.60 V vs. RHE) at different scan rates of 20, 40, 60, 80, and 100 mV s^-1^ and (b) linear fitting of the capacitive current as a function of CV scan rate for Zn-NiMo/NF_L.


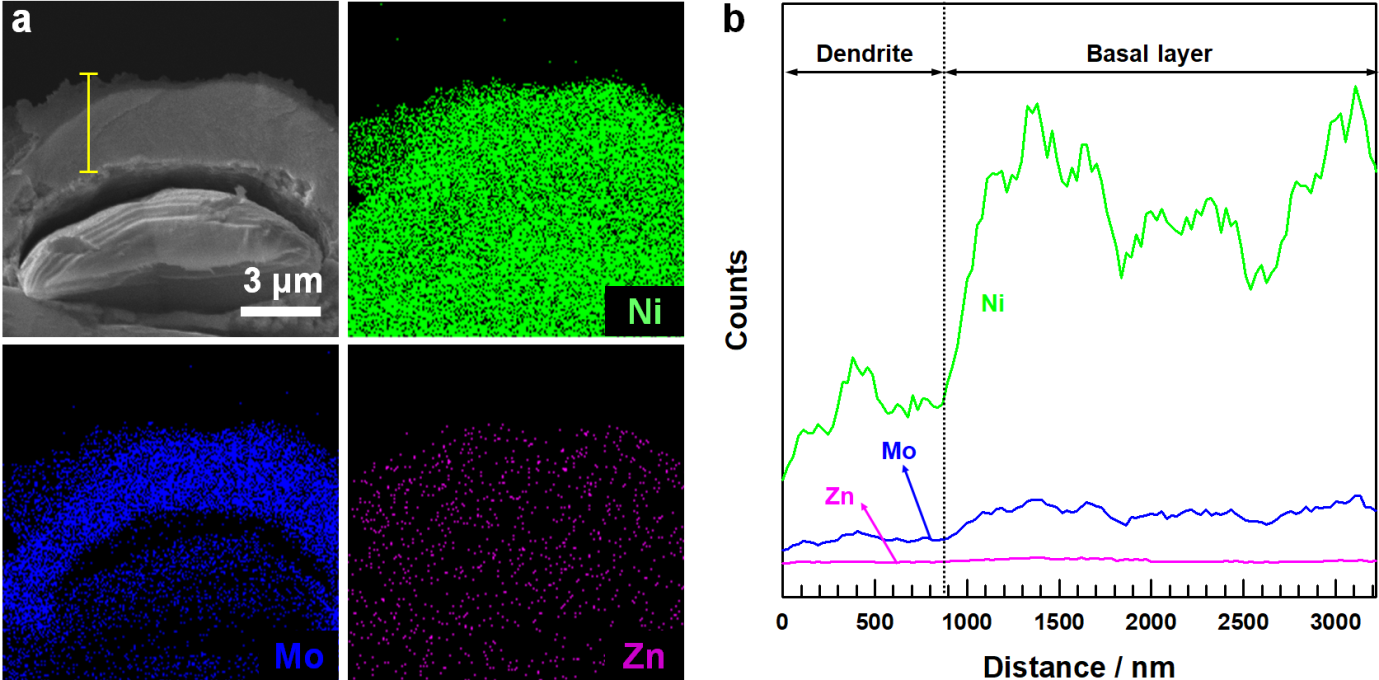


**Figure S10.** (a) Edge view SEM-EDS mapping of Zn-NiMo/NF_L and (b) corresponding line scanning profile.


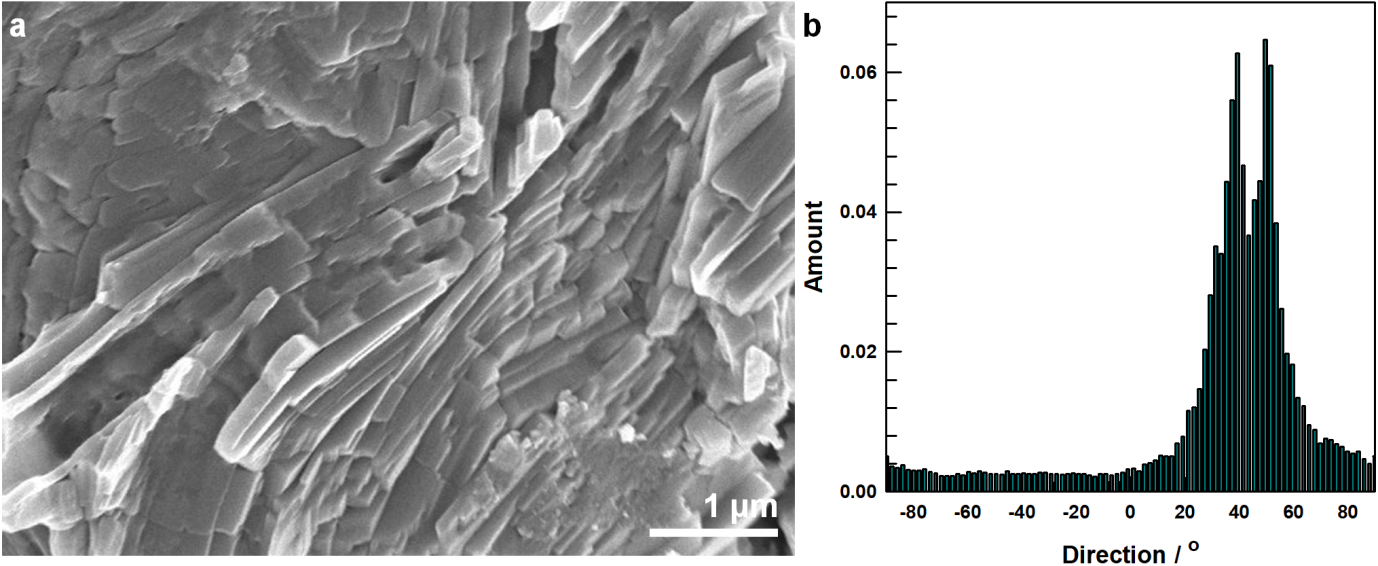


**Figure S11.** Spot 1: (a) SEM image of the reconstructed NiMo-rich dendritic structure. (b) Corresponding directionality histogram from Fiji software.

**
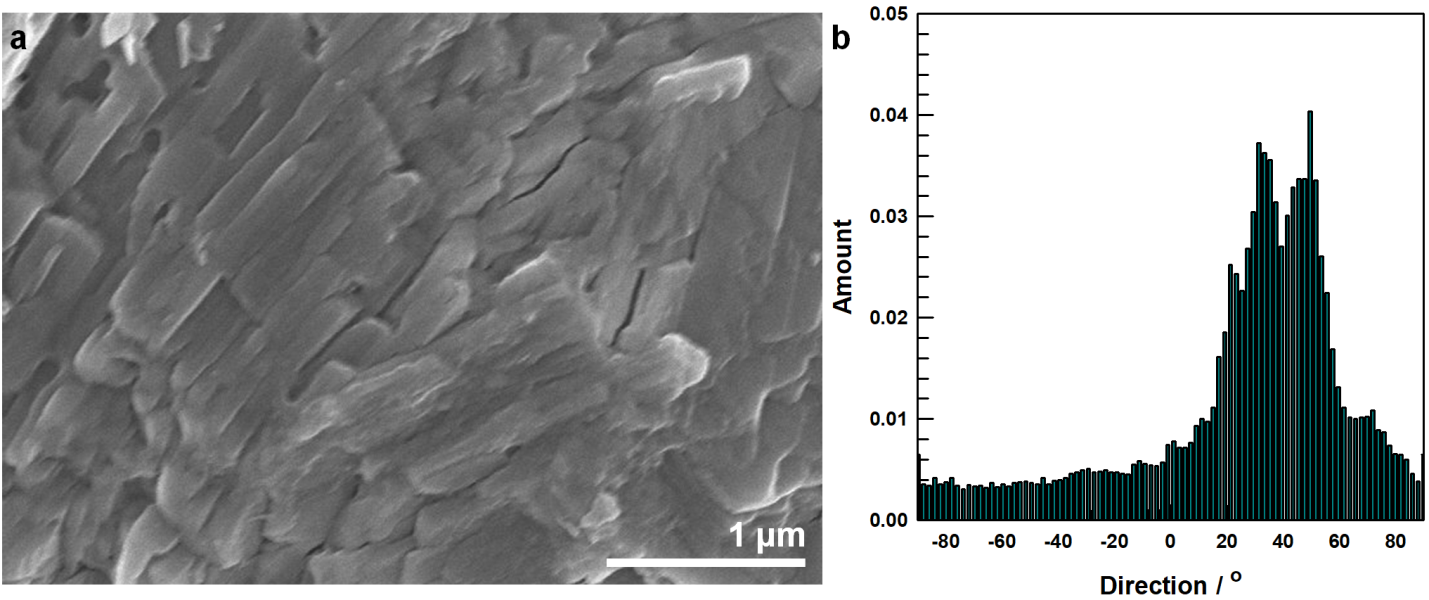
**

**Figure S12**. Spot 2: (a) SEM image of the reconstructed NiMo-rich dendritic structure. (b) Corresponding directionality histogram from Fiji software.


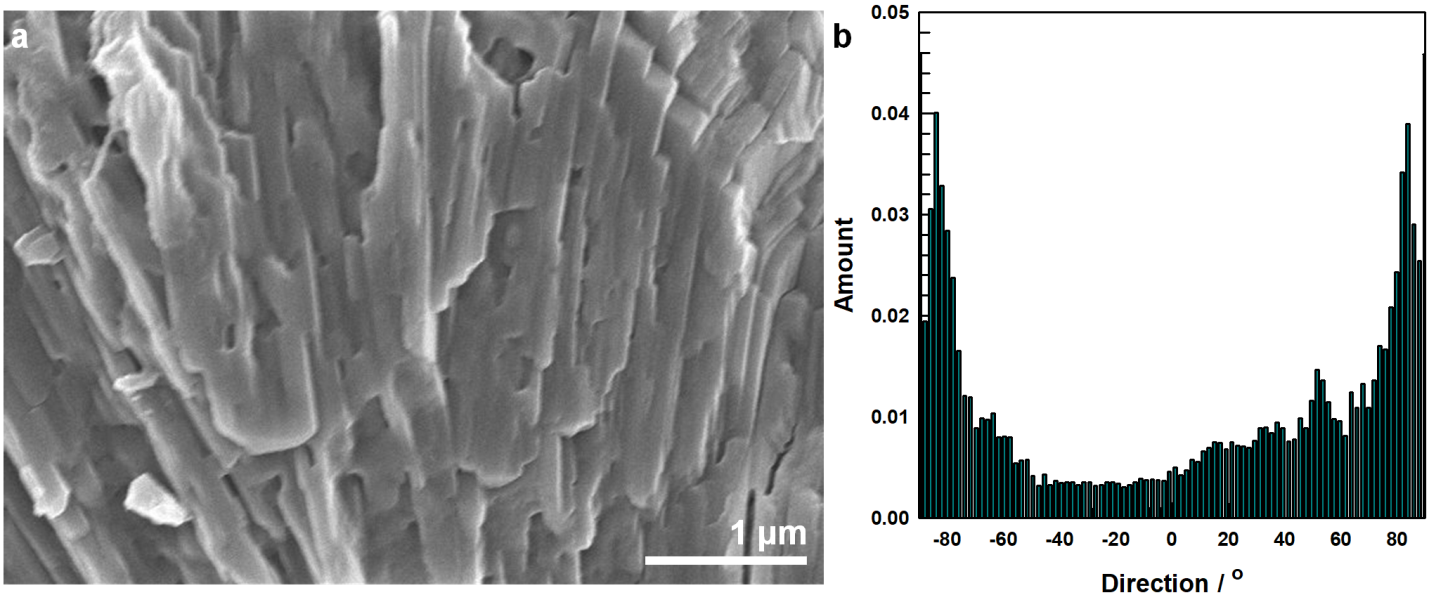


**Figure S13.** Spot 3: (a) SEM image of the reconstructed NiMo-rich dendritic structure. (b) Corresponding directionality histogram from Fiji software.


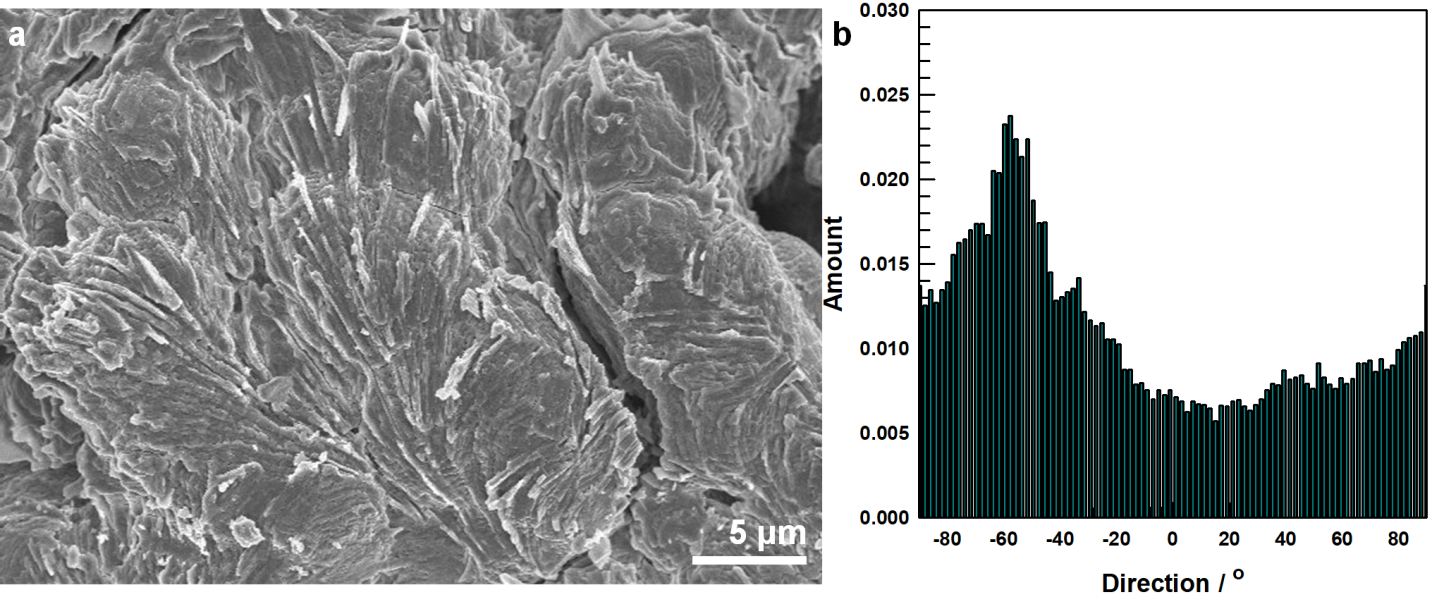


**Figure S14.** Spot 4: (a) SEM image of the reconstructed NiMo-rich dendritic structure. (b) Corresponding directionality histogram from Fiji software.


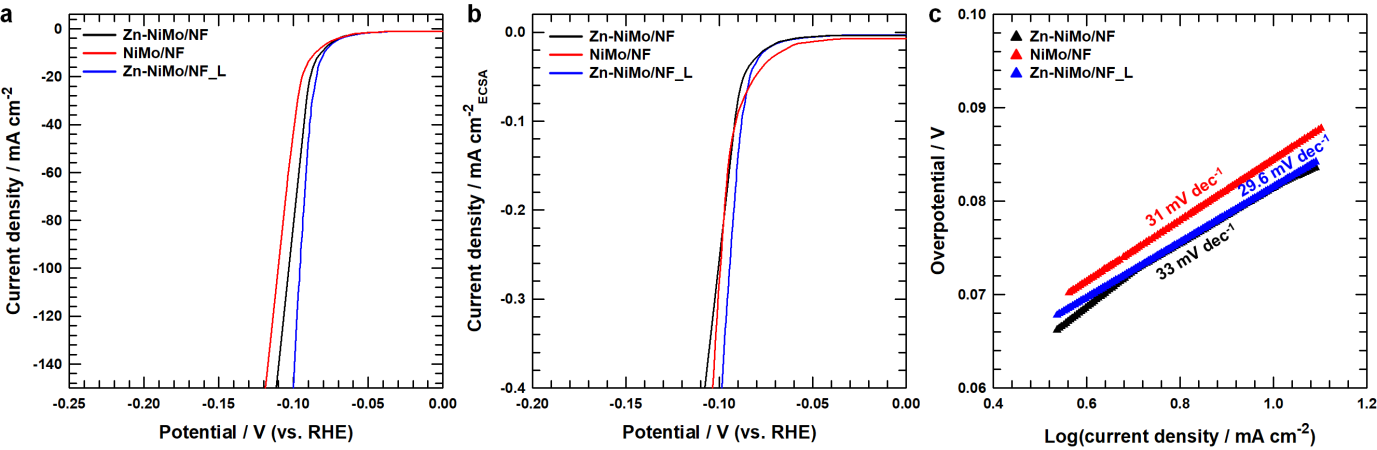


**Figure S15.** (a) iR-corrected Linear sweep voltammetry (LSV) curves, (b) ECSA-normalized LSV curves, and (c) Tafel plots for Zn-NiMo/NF, NiMo/NF, and Zn-NiMo/NF_L.


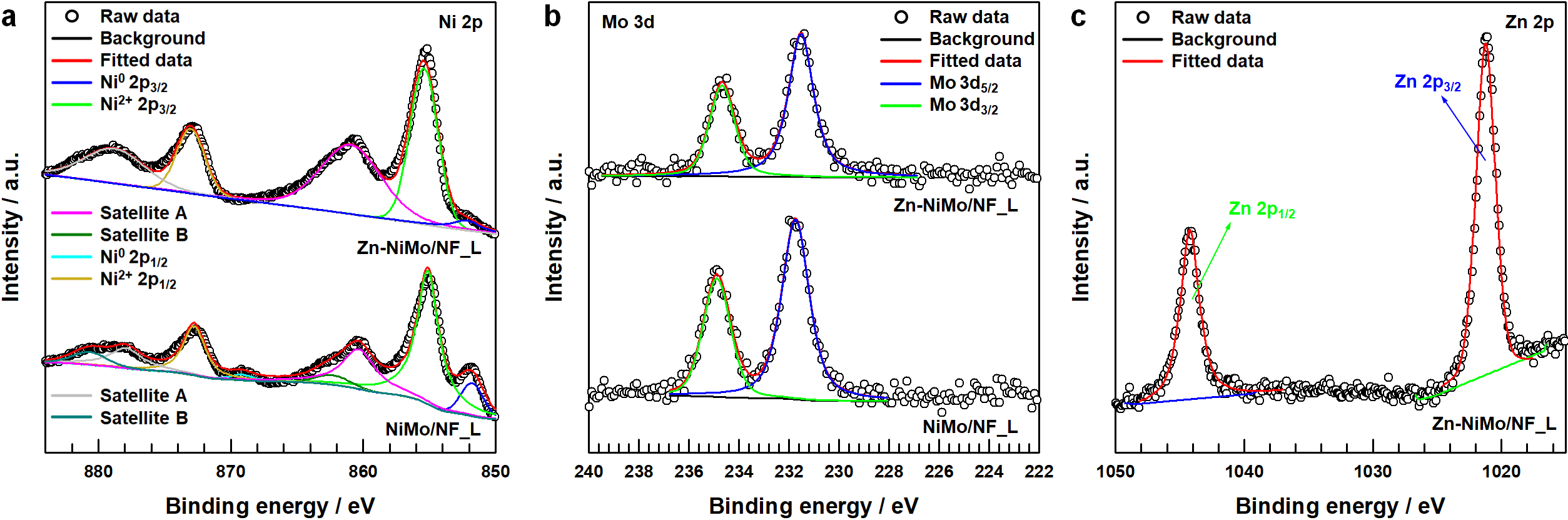


**Figure S16.** X-ray photoelectron spectroscopy (XPS) spectra after load fluctuation (-150/0 mA cm^-2^). (a) Ni 2p and (b) Mo 3d spectra of Zn-NiMo/NF_L and NiMo/NF_L. (c) Zn 2p spectra of Zn-NiMo/NF_L. Both catalysts retain similar electronic structures of Mo and Zn after load fluctuation test.


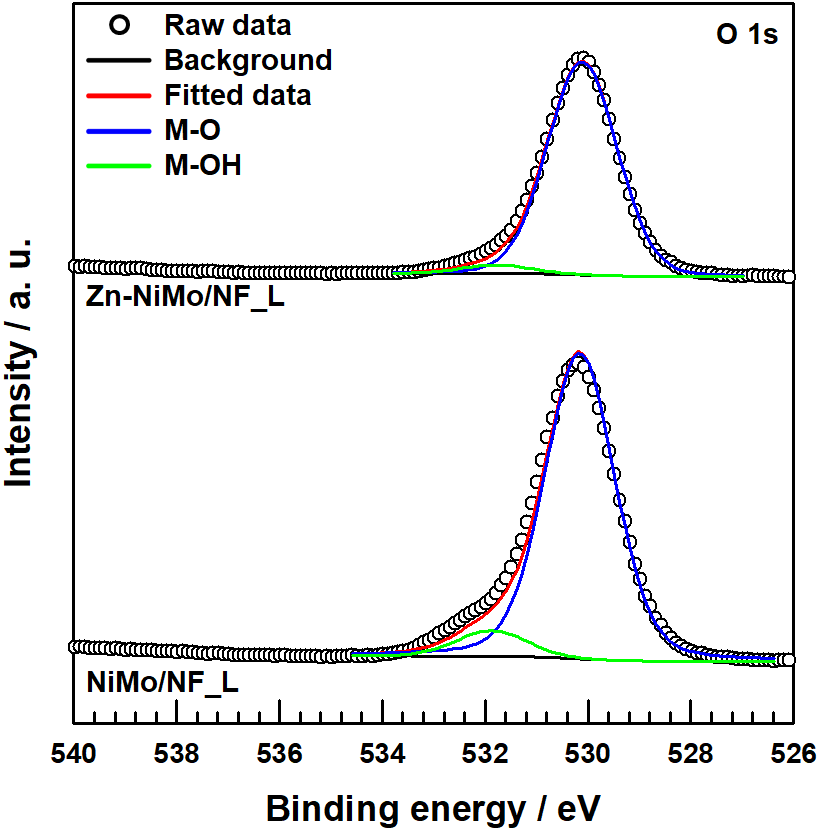


**Figure S17.** XPS O 1s spectra of Zn-NiMo/NF_L and NiMo/NF_L.


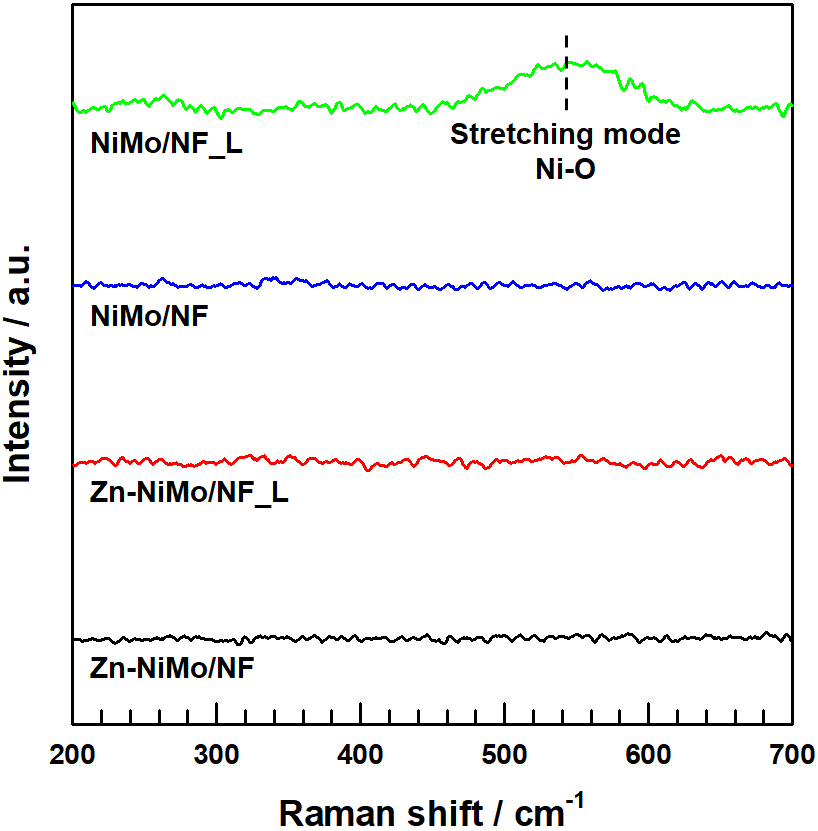


**Figure S18.** Raman spectra (200-700 cm^-1^) of Zn-NiMo/NF and NiMo/NF before and after load fluctuation (-150/0 mA cm^-2^).


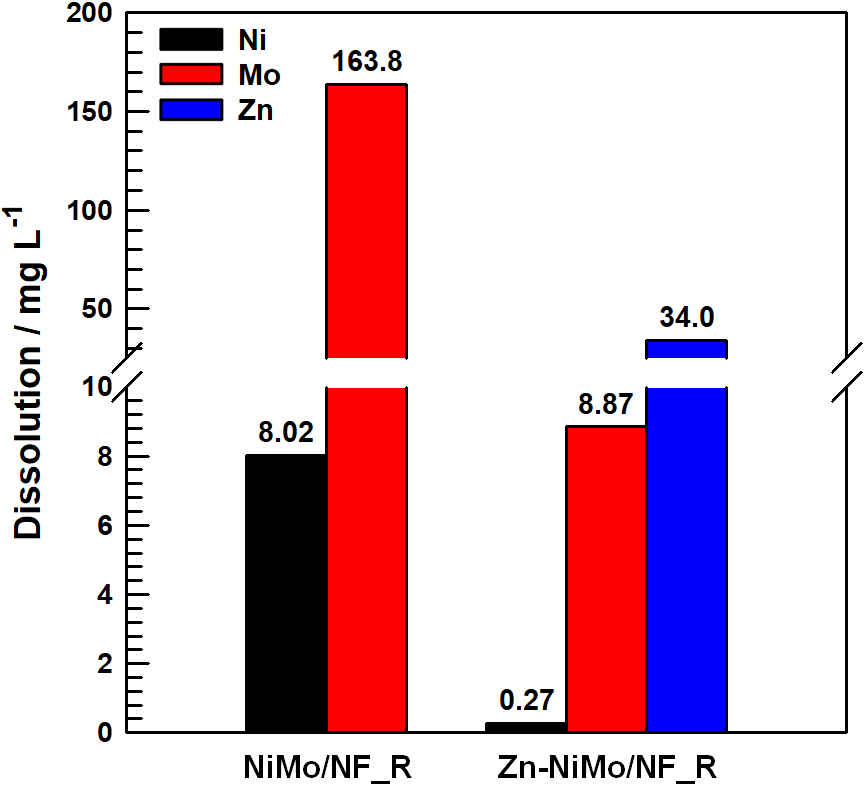


**Figure S19.** Comparison of the metal dissolution amount from NiMo/NF and Zn-NiMo/NF after load fluctuation (-500/50 mA cm^-2^).

**
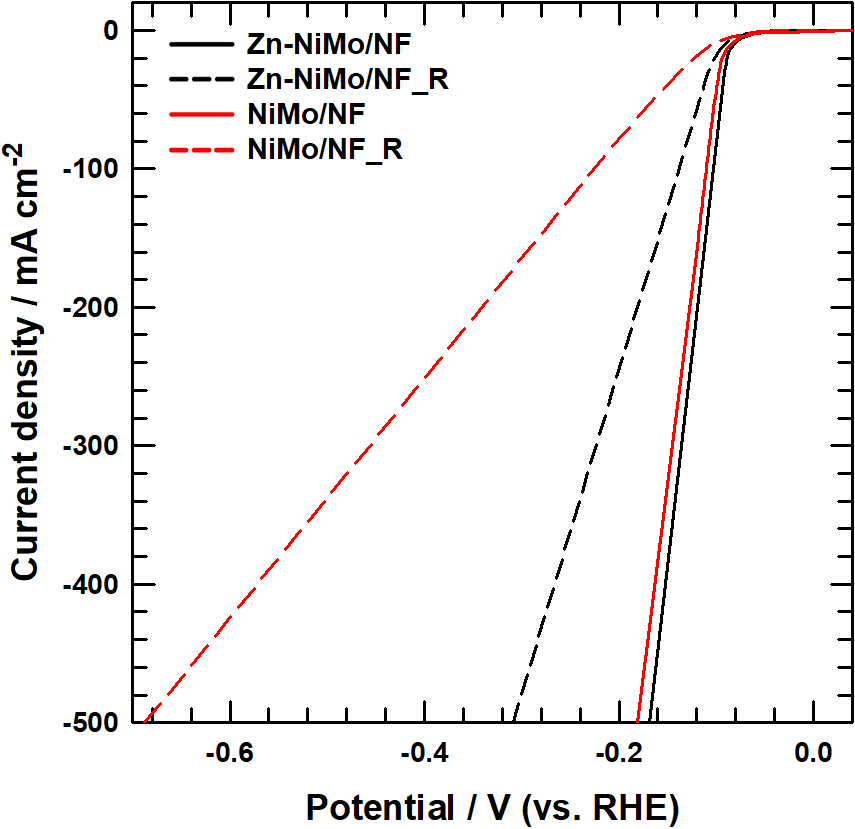
**

**Figure S20.** Linear sweep voltammetry (LSV) curves of Zn-NiMo/NF and NiMo/NF before and after load fluctuation (-500/50 mA cm^-2^).


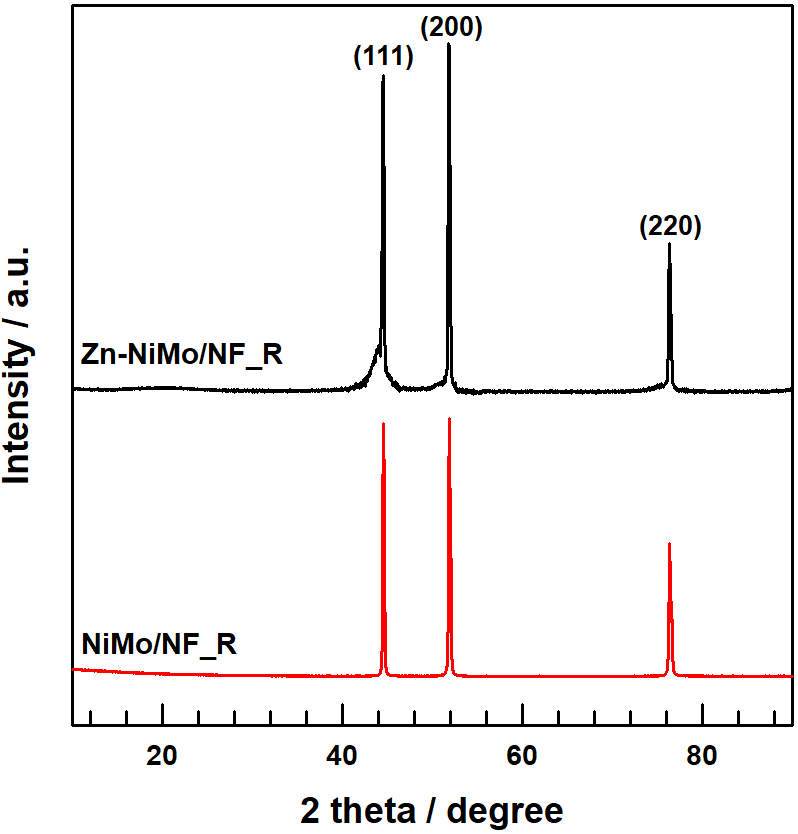


**Figure S21.** X-ray diffraction (XRD) patterns of Zn-NiMo/NF_R and NiMo/NF_R.


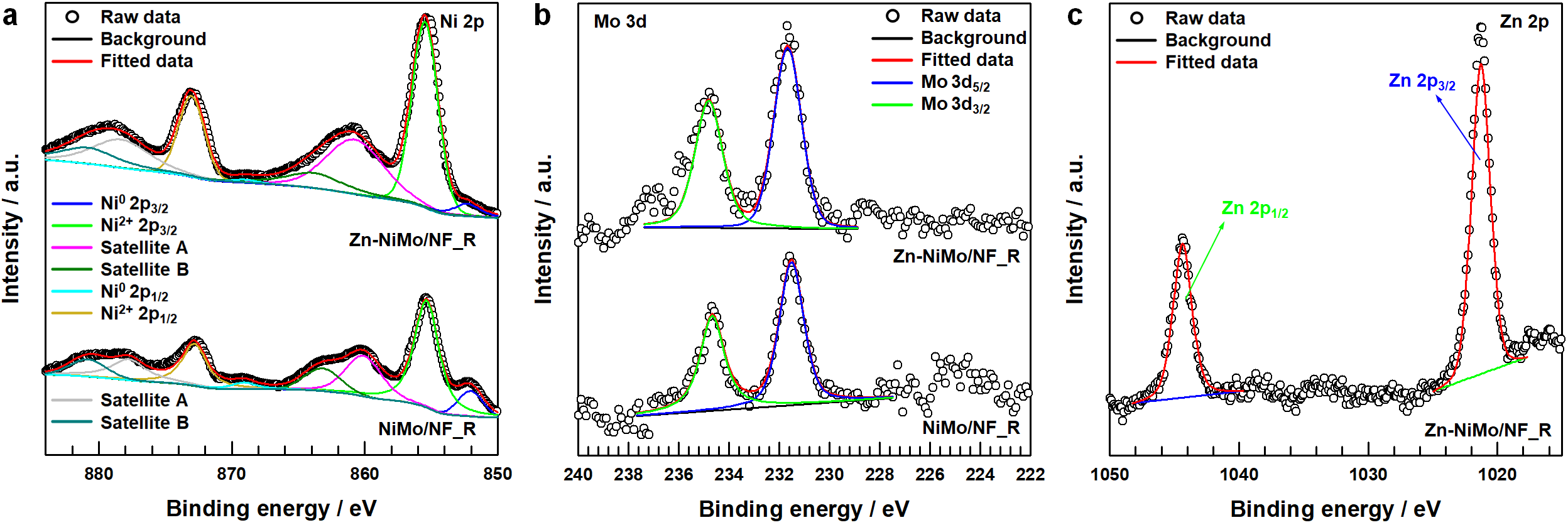


**Figure S22.** (a) XPS Ni 2p and (b) XPS Mo 3d spectra of Zn-NiMo/NF_R and NiMo/NF_R. (c) XPS Zn 2p spectra of Zn-NiMo/NF_R. Both catalysts maintain similar electronic structures of Mo and Zn after reverse current-mimicked load fluctuation test.


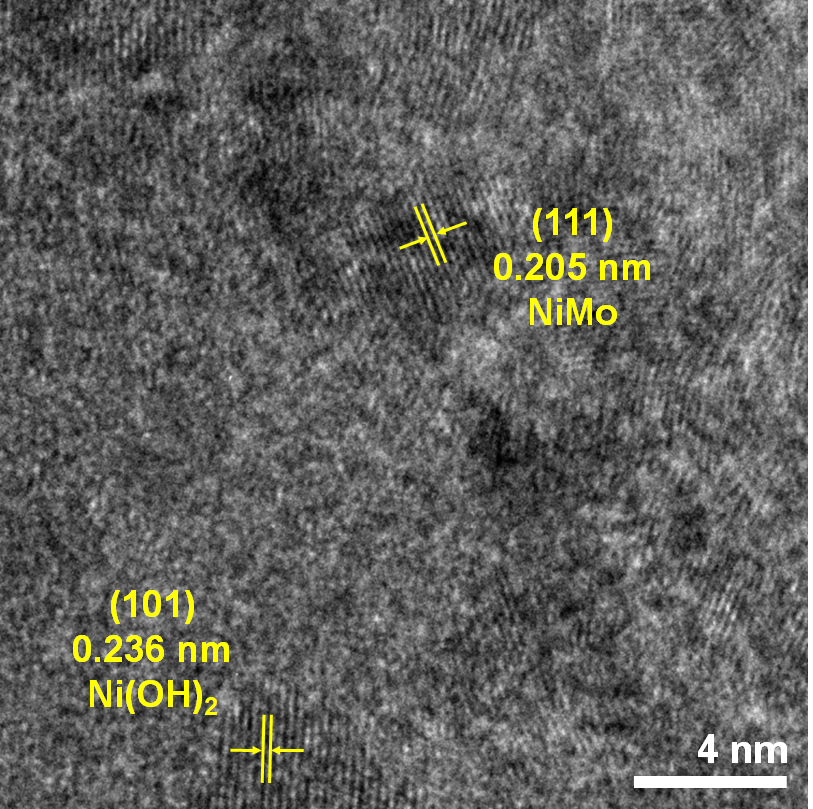


**Figure S23.** Lattice structures of NiMo/NF_R examined by HR-TEM.


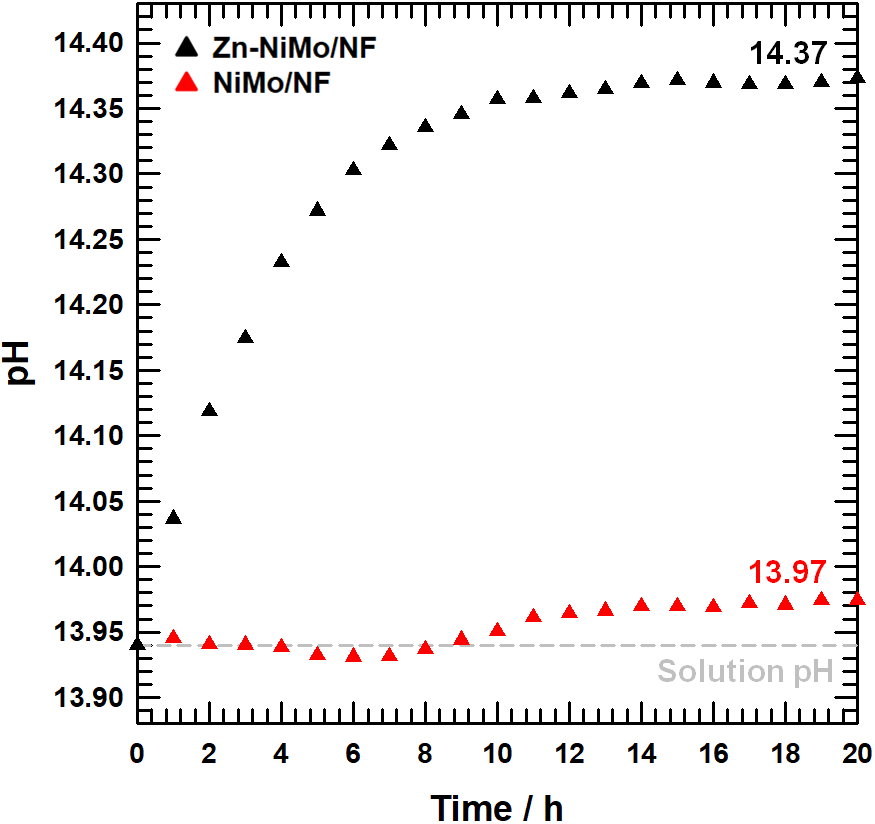


**Figure S24**. Electrode local pH of the Zn-NiMo/NF and NiMo during load fluctuation (-500/50 mA cm^-2^).


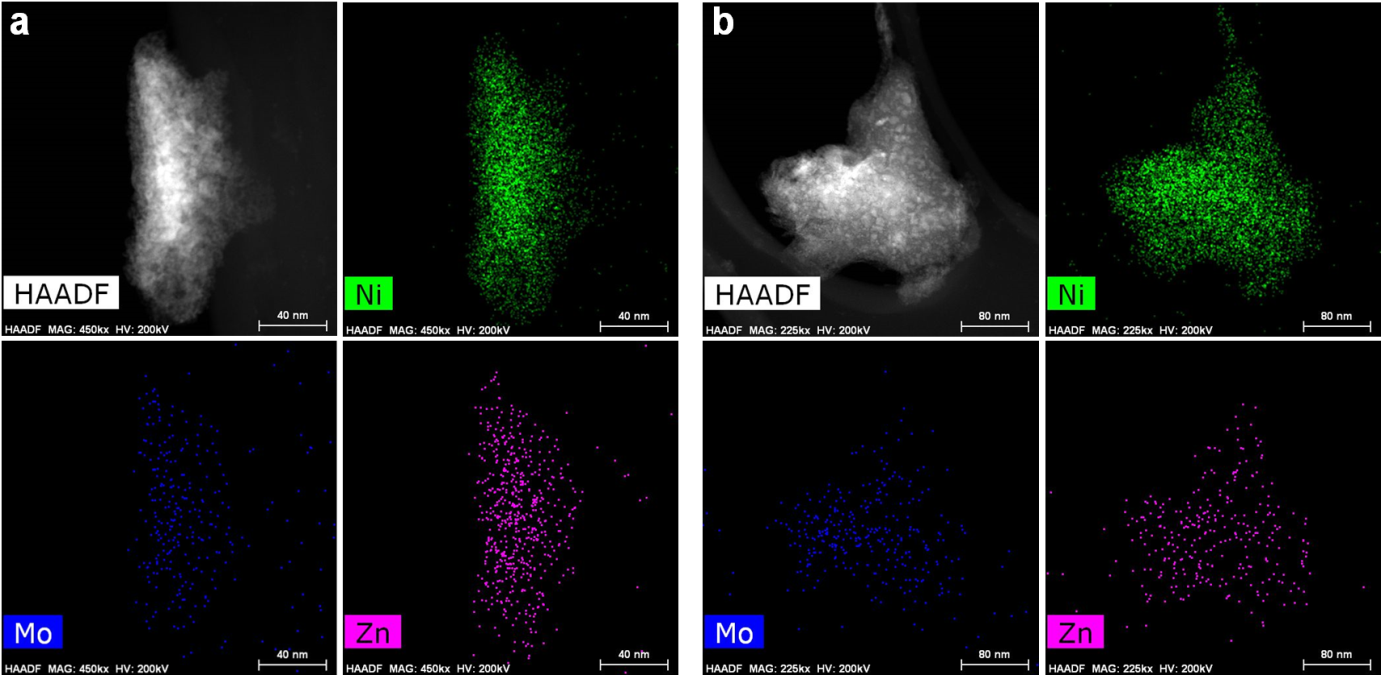


**Figure S25.** TEM-EDS mapping of (a) Zn-NiMo/NF_R (half-cell test, -500/50 mA cm^-2^, 100h) and (b) Zn-NiMo/NF after AEMWE full-cell test (1000/0 mA cm^-2^, 100h)

**Table S1.** Weight ratio percentage (wt.%) of elements in the samples.

| **Sample** | **Ni**  **(wt.%)** | **Mo**  **(wt.%)** | **Zn**  **(wt.%)** |
| --- | --- | --- | --- |
| **NiMo/NF** | 86.6 | 13.4 | - |
| **Zn-NiMo/NF** | 82.6 | 12.8 | 4.6 |

The Ni, Mo, and Zn content (wt.%) in the samples was determined using inductively coupled plasma optical emission spectroscopy (ICP-OES).

**Table S2.** HER activity of Zn-NiMo/NF compared to other reported state-of-the art electrocatalysts,

| **HER electrocatalysts** | **Electrolyte** | **Overpotential (mV)**  **(at j = 50 mA cm^-2^)** | **Ref** |
| --- | --- | --- | --- |
| **Ru/NiMnB** | 1.0 M KOH | 103 | [2] |
| **Ce/CoP-Ni_3_P** | 1.0 M KOH | 105 | [3] |
| **NiFeMo** | 1.0 M KOH | 350 | [4] |
| **NiSe/Ni_3_Se_4_** | 1.0 M KOH | 210 | [5] |
| **NiCu/Co** | 1.0 M KOH | 195 | [6] |
| **NiFeRu** | 1.0 M KOH | 115 | [7] |
| **NiMo_450_/CoP** | 1.0 M KOH | 150 | [8] |
| **NMSeS/NSe** | 1.0 M KOH | 151 | [9] |
| **Ni-B-P** | 1.0 M KOH | 90 | [10] |
| **Mo/NiCoP** | 1.0 M KOH | 165 | [11] |
| **Zn-NiMo/NF** | 1.0 M KOH | 94.6 | This work |

**Table S3.** ICP-MS data of electrolyte (1.0 M KOH) after 10 h of load fluctuation at -150/0 mA cm^-2^.

| **Sample** | **Ni**  **(μg L^-1^)** | **Mo**  **(μg L^-1^)** | **Zn**  **(μg L^-1^)** |
| --- | --- | --- | --- |
| **NiMo/NF_L** | 265 | 5,440 | - |
| **Zn-NiMo/NF_L** | 7.5 | 231.5 | 4,820 |

**Table S4.** ICP-MS data of electrolyte (1.0 M KOH) after 100 h of load fluctuation at -500/50 mA cm^-2^.

| **Sample** | **Ni**  **(mg L^-1^)** | **Mo**  **(mg L^-1^)** | **Zn**  **(mg L^-1^)** |
| --- | --- | --- | --- |
| **NiMo/NF_R** | 8.02 | 163.8 | - |
| **Zn-NiMo/NF_R** | 0.27 | 8.87 | 34.0 |

To further elucidate the elemental dissolution behavior under harsh load fluctuation conditions, inductively coupled plasma mass spectrometry (ICP-MS) analysis was conducted on the post-electrolysis electrolyte. Zn-NiMo/NF sample exhibited predominant Zn leaching (34 mg L^-1^), accompanied by minimal Ni and Mo dissolution (0.27 and 8.87 mg L^-1^, respectively). In contrast, the NiMo/NF sample showed significantly higher Ni and Mo leaching (8.02 and 163.8 mg L^-1^, respectively).

**Table S5.** AEMWE performance of Zn-NiMo/NF compared to other reported state-of-the art electrocatalysts,

| **Cathode catalyst** | **Anode catalyst** | **Electrolyte** | **Membrane** | **Temperature**  **(°C)** | **Activity** | | **Ref** |
| --- | --- | --- | --- | --- | --- | --- | --- |
| **NiFeOH_x_/NF** | **Ru SAs/WC_x_** | 1.0 M KOH | Sustainion X37-50 | 80 | 1.0 A cm^-2^ at 1.79 V | | [12] |
| **Ni-Fe LDH** | **Ni_3_Mo** | 1.0 M KOH | Sustainion X37-50 | 50 | 1.0 A cm^-2^ at 1.82 V | | [13] |
| **B, V-Ni_2_P** | **NiFeOOH** | 1.0 M KOH | Sustainion X37-50 | 55 | 1.0 A cm^-2^ at 1.92 V | | [14] |
| **NiMoO_x_@CMK-3** | **NiFe LDH** | 1.0 M KOH | Fumasep, FAA-3–50 | 25 | 1.0 A cm^-2^ at 1.965 V | | [15] |
| **Pt_SA_–Mn,Fe–Ni LDHs** | **Pt_SA_–Mn,Fe–Ni LDHs** | 1.0 M KOH | Sustainion X37-50 | 60 | 1.0 A cm^-2^ at 1.97 V | | [16] |
| **Fe_2_P_2_S_6_ NCs** | **Fe_2_P_2_S_6_ NCs** | 1.0 M KOH | YAB, Foma Corporation | 50 | 0.37 A cm^-2^ at 1.8 V | [17] | |
| **CoVO@NF** | **CoVO@NF** | 1.0 M KOH | Fumasep FAA-3-PK-130 | 70 | 0.5 A cm^-2^ at 1.76 V | [18] | |
| **NiMoN_x_** | **Fe-NiMoN_x_** | 1.0 M KOH | Sustainion X37-50 | 80 | 1.0 A cm^-2^ at 1.57 V | [19] | |
| **P-Os/NiFe** | **RuO_2_/NiFe** | 1.0 M KOH | Sustainion X37-50 | 60 | 1.0 A cm^-2^ at 2.02 V | [20] | |
| **Ni-XG/KB** | **NiFe/Ni-foam** | 1.0 M KOH | PiperION A40-HCO3 | 80 | 1.0 A cm^-2^ at 1.90 V | [21] | |
| **Zn-NiMo/NF** | **IrO_2_/NF** | 1.0 M KOH | Sustainion X37-50 | 60 | 1.0 A cm^-2^ at 1.645 V | This work | |

**References**

1. Z. Zhuang, Y. Wang, J. Pei, Q. Dang, X. Wang, X. Zhang, X. Guo, M. Chen, J. Wang, H. Li, W. Zhu, **2025**.
2. M. A. Habib, S. Lin, M. H. Joni, S. A. Dristy, R. Mandavkar, J.-H. Jeong, J. Lee, *J. Energy Chem.* **2025**, *100*, 397.
3. F. Zhang, X. Wang, W. Han, Y. Qian, L. Qiu, Y. He, L. Lei, X. Zhang, *Adv. Funct. Mater.* **2023**, *33* (9), 2212381.
4. Z. Wang, H. Chen, J. Bao, Y. Song, X. She, G. Lv, J. Deng, H. Li, H. Xu, *Appl. Surf. Sci.* **2023**, *607*, 154803.
5. L. Tan, J. Yu, H. Wang, H. Gao, X. Liu, L. Wang, X. She, T. Zhan, *Appl. Catal., B* **2022**, *303*, 120915.
6. A. Kumar, S. K. Purkayastha, A. K. Guha, M. R. Das, S. Deka, *ACS Catal.* **2023**, *13* (16), 10615.
7. Y. Wang, Q. Ye, L. Lin, Y. Zhao, Y. Cheng, *J. Colloid Interface Sci.* **2023**, *651*, 1008.
8. L. Luo, H. Li, S. Liu, *Fuel* **2025**, *399*, 135675.
9. M. Fan, L. Yu, L. Gao, L. Cui, Z. Dou, *Int. J. Hydrogen Energy* **2025**, *168*, 150548.
10. M. A. Habib, R. Mandavkar, S. Lin, S. Burse, T. Khalid, M. H. Joni, J.-H. Jeong, J. Lee, *Chem. Eng. J.* **2023**, *462*, 142177.
11. J. Jiang, R. Sun, X. Huang, W. Xu, S. Zhou, Y. Wei, S. Han, Y. Li, *Compos. B: Eng.* **2023**, *263*, 110834.
12. X. Lin, W. Hu, J. Xu, X. Liu, W. Jiang, X. Ma, D. He, Z. Wang, W. Li, L.-M. Yang, H. Zhou, Y. Wu, *J. Am. Chem. Soc.* **2024**, *146* (7), 4883.
13. W. Lee, H. Yun, Y. Kim, S. S. Jeon, H. T. Chung, B. Han, H. Lee, *ACS Catal.* **2023**, *13* (17), 11589.
14. T. Zhao, S. Wang, C. Jia, C. Rong, Z. Su, K. Dastafkan, Q. Zhang, C. Zhao, *Small* **2023**, *19* (27), 2208076.
15. Y. Chen, K. Yue, J.-W. Zhao, Z. Cai, X. Wang, Y. Yan, *Chem. Eng. J.* **2023**, *466*, 143097.
16. K. D. Tran, T. H. Nguyen, D. T. Tran, V. A. Dinh, N. H. Kim, J. H. Lee, *ACS nano* **2024**, *18* (25), 16222.
17. J. Chang, G. Wang, A. Belharsa, J. Ge, W. Xing, Y. Yang, *Small Methods* **2020**, *4* (6), 1900632
18. Z. Liang, D. Shen, Y. Wei, F. Sun, Y. Xie, L. Wang, H. Fu, *Adv. Mater.* **2024**, *36* (41), 2408634.
19. P. Chen, X. Hu, *Adv. Energy Mater.* **2020**, *10* (39), 2002285.
20. Q. Li, X. Fu, H. Li, Z. Xiao, G. Xu, D. Chen, C. Li, W. Jin, T. Ma, Z. Wu, L. Wang, S. Feng, *Adv. Funct. Mater.* **2024**, *34* (48), 2408517.
21. D. M. Sayed, L. Osmieri, H. Yu, L. Amichi, H. M. Meyer Iii, J. D. Jernigen, P. Zelenay, *ACS Appl. Energy Mater.* **2025**, *8* (15), 10949.
